# Noncatalytic surface electrostatic networks tune thermolability in uracil-DNA glycosylase

**DOI:** 10.1016/j.jbc.2026.113212

**Published:** 2026-05-28

**Authors:** Rita S.M. Simões, João S. Teodoro, Victor D. Alves, Ana Luísa Carvalho, Carlos M.G.A. Fontes, Pedro Bule

**Affiliations:** 1 NZYtech – Genes & Enzymes, Campus do Lumiar, Lisbon, Portugal; 2 CIISA – Centre for Interdisciplinary Research in Animal Health, Faculty of Veterinary Medicine, University of Lisbon, Lisbon, Portugal; 3 Associate Laboratory for Animal and Veterinary Sciences (AL4AnimalS), Lisbon, Portugal; 4 UCIBIO, Chemistry Department, NOVA School of Science and Technology, Universidade NOVA de Lisboa, Caparica, Portugal; 5 Associate Laboratory i4HB – Institute for Health and Bioeconomy, NOVA School of Science and Technology, Universidade NOVA de Lisboa, Caparica, Portugal

**Keywords:** uracil-DNA glycosylase, carryover contamination, psychrophilic enzymes, thermostability, thermolability, electrostatics, enzyme structure, enzyme inactivation, enzyme mutation, site-directed mutagenesis

## Abstract

Uracil-DNA glycosylases (UDGs) are widely used to prevent carryover contamination in nucleic acid amplification–based diagnostics; however, existing thermolabile UDGs exhibit limited thermal inactivation windows for emerging applications. Here, we combine evolutionary mining, structural analysis, and structure-guided saturation mutagenesis to define non-catalytic determinants that tune UDG thermolability without compromising catalytic function. From 8482 bacterial UDG sequences, we assembled a 24-member diversity panel and identified UDG_7 as a naturally thermolabile scaffold coupling robust low-temperature activity with sharp inactivation near 45 °C. The crystal structure of UDG_7 reveals a canonical family-I α/β fold with a fully conserved active site, closely resembling both mesophilic human and *Escherichia coli* UDGs and thermolabile cod UDG. These structural insights guided the design of a single-site variant library targeting 48 non-catalytic positions involved in packing and electrostatic networks. Pooled thermal shift assays distinguished a rigid structural core from 16 surface thermolability hotspots. A high-throughput functional screening of 480 single mutants yielded 114 clones with a desirable “on–off–off” profile and, after sequence consolidation, identified 54 unique variants that retained activity at 25 °C but lost activity at 30 to 37.5 °C. Biochemical characterization revealed nine single substitutions, Q51I, T112Y, V144M, D167F, R201F, R201Y, D219M, R221P, and R221D, that markedly lower the melting temperature while preserving near-native activity. Together, these results indicate that UDG_7 thermolability is encoded by a distributed, surface-biased electrostatic network that can be selectively disrupted without perturbing the conserved catalytic core, shifting the functional inactivation boundary downward and supporting robust carryover control under low-temperature amplification constraints.

Nucleic acid amplification-based assays have expanded the scope of molecular diagnostics by enabling rapid, highly sensitive, and specific detection of diverse clinically relevant DNA and RNA targets (1). However, the extraordinary amplification efficiency of polymerase chain reaction (PCR) also renders these assays acutely susceptible to carryover contamination, a well-known source of false-positive results in both laboratory and point-of-care (POC) setting (2, 3, 4, 5). A widely adopted biochemical safeguard is the dUTP/uracil-DNA glycosylase (UDG) system, in which dTTP is partially replaced by dUTP so that all amplification products incorporate uracil and any contaminating amplicons are selectively degraded by UDG before amplification begins (6, 7, 8). UDG (also termed UNG) is a monofunctional base-excision repair enzyme that cleaves the N-glycosidic bond of uracil in DNA, generating abasic sites that block further extension by DNA polymerases (6, 7).

For effective carryover prevention, the ideal UDG must efficiently remove uracilated DNA during reaction assembly (0–4 °C) or at a specific pre-amplification decontamination step (usually at room temperature, 20–25 °C) but then become rapidly and irreversibly inactivated at elevated temperatures, so that newly synthesized amplicons remain intact during PCR amplification (9, 10). Cold-adapted UDGs have therefore emerged as attractive candidates for achieving this “on–off” behavior (11, 12, 13, 14, 15, 16). Atlantic cod UDG (cUNG) is a psychrophilic family I enzyme that displays high catalytic efficiency at low temperature but unfolds at substantially lower temperatures than its human counterpart (14, 17). Comparative structural, thermodynamic, and molecular dynamics studies of Atlantic cod and human UDG have shown that cold adaptation in cUNG involves increased flexibility, enlarged internal cavities, altered ion-pair networks, and a reduced melting temperature, providing a conceptual framework for engineering UDG thermolability (17, 18, 19).

Although cUNG has been widely adopted in commercial dUTP/UDG systems as a benchmark thermolabile enzyme (14), emerging diagnostic workflows increasingly operate within narrow and comparatively low temperature ranges, in which a clear separation between the UDG “on” step (sample handling and pre-incubation, typically ≤20–25 °C) and the amplification step, during which UDG must be inactivated, becomes difficult. This is especially important when molecular diagnostics combine reverse transcription and amplification in a single tube—such as in RT-qPCR—or when they rely on moderate to low-temperature isothermal methods (20, 21, 22) such as loop-mediated amplification (LAMP), helicase-dependent amplification (HDA), or recombinase polymerase amplification (RPA), for example. Unlike PCR, these workflows typically lack an initial high-temperature step, so UDG activity can persist transiently into the stage when target synthesis begins, resulting in degradation of the newly-synthesized dUTP-containing amplification products as they are generated (9). As a response to this constraint, enzyme manufacturers often compensate by recommending the use of lower UDG amounts in the reaction, however this trade-off results in the degradation of only a limited number of contaminating copies per reaction, meaning that high-level contaminations can escape control simply because enzyme concentration cannot be increased without risking incomplete inactivation (9). Together, these constraints motivate the development of a systematic strategy to engineer UDGs with more aggressively shifted and better-defined inactivation boundaries while retaining strong low-temperature activity.

Temperature-sensitive (Ts) mutant variants have long been used to regulate enzyme activity both *in vitro* and *in vivo*. Early research on glutathione transferases, λ-repressor, lysozymes, and other model proteins demonstrated that helix-capping motifs, local hydrogen-bond networks, and buried hydrophobic residues are prime targets for tuning stability with minimal impact on activity. (23, 24, 25). These principles have since been distilled into computational tools such as TSpred, which predicts candidate Ts positions from residue depth and hydrophobicity (26, 27, 28, 29). For UDGs, however, rational engineering of thermolability remains comparatively underexplored. Recently, Park and colleagues demonstrated that disruption of interaction hubs in *Escherichia coli* UDG, guided by large-scale molecular dynamics, interaction-energy network analysis, and mutagenesis, can produce single-site mutants like D43A (30). These mutants are highly thermolabile but still maintain strong activity at low temperatures and perform well in PCR carryover-control assays. (30). While these results highlight the feasibility of engineering diagnostically useful thermolabile UDGs, they focus on a single mesophilic scaffold and a limited set of positions, leaving open how broader non-catalytic networks encode UDG thermolability and how much this property can be adjusted without compromising activity. Additionally, they do not fully utilize the structural knowledge of UDGs that map the catalytic machinery and five conserved family-I motifs involved in base flipping, uracil recognition, and backbone compression. (19, 26, 31, 32, 33). These structural and computational studies suggest that UDG thermolability is encoded not within the catalytic core but in distributed, non-catalytic interactions that modulate global stability.

Here, we combine evolutionary sequence mining, structural analysis, and targeted saturation mutagenesis to systematically investigate the non-catalytic determinants of UDG thermolability in a diagnostic context. By integrating concepts from cold-adapted enzymology, Ts-mutant design, and UDG structural biology, our work establishes a general strategy to engineer next-generation thermolabile UDGs with tunable inactivation windows for emerging low-temperature molecular diagnostics applications.

## Results

### Phylogeny-guided construction of a 24-member UDG diversity panel

**Figure 1:**
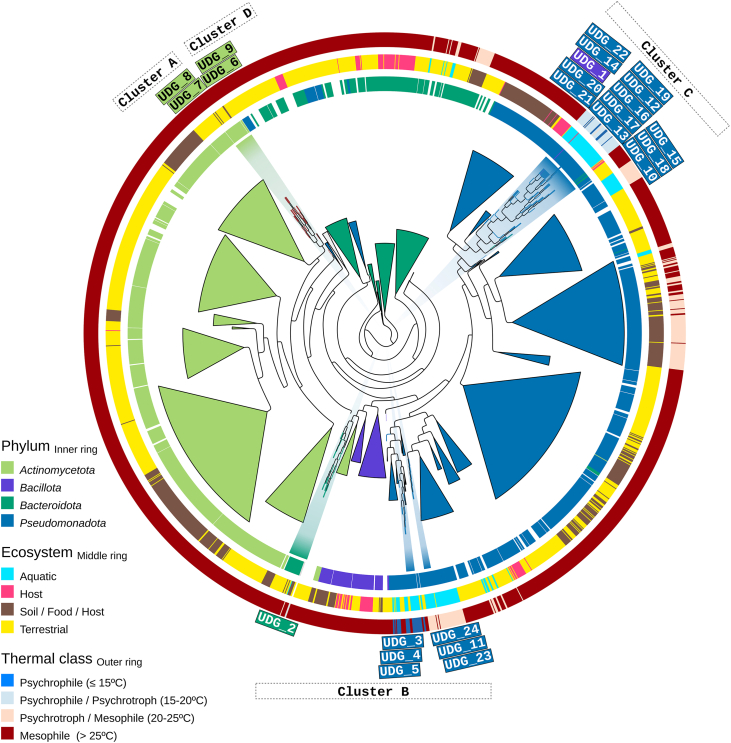
**Phylogenetic landscape of bacterial family-I UDGs and selection of the 24-member panel.** Maximum-likelihood tree of a non-redundant set representing 8482 bacterial UDG sequences. The inner ring reports phylum-level taxonomy (Actinomycetota, Bacillota, Bacteroidota, Pseudomonadota, representing the preponderant taxonomic groups), the middle ring indicates the predominant ecosystem of the source organism (aquatic, host-associated, soil/food/host, terrestrial), and the outer ring encodes thermal class (psychrophile, psychrophile/psychrotroph, psychrotroph/mesophile, mesophile). Semi-translucent wedges highlight major cold-adapted clades, from which 24 representative enzymes (UDG_1–UDG_24) were chosen to capture deep phylogenetic branches, ecological breadth, and an enrichment in low-temperature lineages. Labels mark the positions of these selected UDGs on the tree, colored according to phylum-level taxonomy, and cluster identification (A-D) based on Fig. S1.

To explore natural sequence determinants of UDG thermolability, we assembled a curated database of 8482 UDG sequences spanning a broad taxonomic range of bacteria adapted to psychrophilic, psychrotrophic, and mesophilic environments. Sequence clustering and phylogenetic analyses revealed a highly diverse sequence space with multiple deeply branching lineages (Fig. 1). From this landscape, we selected 24 representative enzymes (UDG_1 to UDG_24) chosen to span deep phylogenetic branches and contrasting ecological niches, with an intentional bias toward cold-adapted lineages predicted to encode thermolabile UDGs (Fig. S1). The resulting panel spans eight bacterial genera isolated from marine, permafrost, soil, food-associated, and clinical environments. It includes enzymes from typical psychrophilic taxa, such as *Colwellia* and *Psychrobacter* (growth temperatures of 0–10 °C), as well as from low-temperature-adapted and classical mesophilic species (Table S1). Pairwise sequence identities among the 24 UDGs range from approximately 38% to 98%, underscoring substantial sequence divergence, yet the phylogeny resolves them into four well-supported clusters (A–D) of closely related enzymes (Fig. S1).

Despite broad overall divergence, multiple-sequence alignment of the 24 UDGs together with the structurally characterized *E. coli* and Atlantic cod UDGs shows that the canonical family-I UDG architecture is strongly conserved, with all five catalytically relevant uracil-excision motifs preserved across the panel (8) (Fig. S2). In motif 1, corresponding to the catalytic water-activating loop, the essential Asp residue is invariant, accompanied by its canonical partner residue (Asp–His in most enzymes and Asp–Thr in UDG_6-UDG_9), with only conservative substitutions in flanking positions. Motif 2, the Pro-rich 5′-compression loop, maintains a conserved Pro-Ser core while surrounding residues differ among enzymes (Leu, Ile, Lys, and Arg). The uracil-binding motif (motif 3) retains essential hydrophobic residues, mainly a sequence of two to three Leu residues and a conserved Asn, with some variation in neighboring hydrogen-bonding partners. In motif 4, the Gly–Ser loop that compresses the 3′ backbone is enriched in small, flexible residues (Gly, Ser, Ala) across all sequences, with modest rearrangements in length and composition among the 24 UDG enzymes. Finally, motif five retains the hallmark Leu intercalation residue responsible for base-flipping; interestingly, UDG_2 carries a Phe at this position, although the hydrophobic nature of the Phe side chain is expected to support a similar base-flipping mechanism prior to catalysis. Significantly, the two key catalytic residues, namely the general base Asp in motif one and the His in motif five that acts as a neutral electrophile, are strictly conserved across all 24 sequences (Fig. S2). Taken together, these patterns indicate that our panel explores extensive sequence variability in peripheral and loop regions while conserving the catalytic core. This combination provides a solid, evolutionarily based framework for analyzing noncatalytic contributions to UDG thermolability and for identifying scaffolds suitable for engineering diagnostically relevant thermolabile variants.

### Recombinant production and functional characterization of the 24-member UDG panel

To assess whether the diversity-guided panel could provide practical scaffolds for engineering a thermolabile UDG, all 24 enzymes were expressed as N-terminally His-tagged proteins in *E. coli* and purified by Ni^2+^-affinity chromatography (representative SDS-PAGE analyses are shown in Fig. S3). Twelve of the 24 enzymes were obtained in soluble form, with yields ranging from approximately 9 to 68 mg L^–1^ and an average yield of 27 mg L^–1^ (Table S2, Fig. S3). UDG_7 and UDG_8 showed the highest soluble expression levels (>50 mg L^–1^), whereas 12 UDGs were largely or fully insoluble under all tested expression conditions, consistent with well-documented solubility challenges of many psychrophilic enzymes when overexpressed in mesophilic *E. coli* hosts (34).

**Figure 2:**
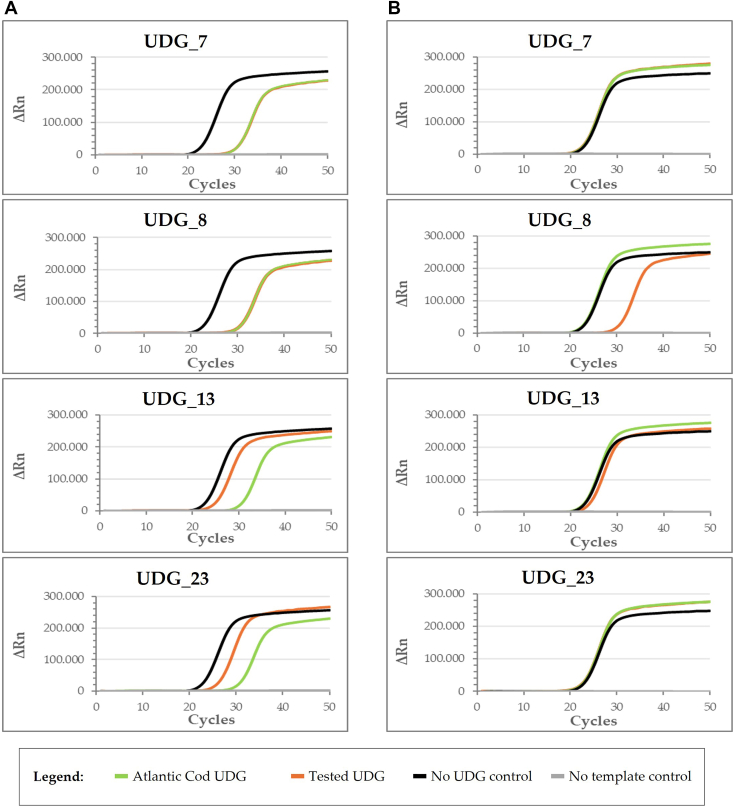
**Real time PCR assays evaluating the performance of selected UDGs.** Real-time PCR amplification curves for 10^5^ copies of a *hPPIA* (*H. sapiens*) template containing 60% dUTP after incubation with UDG_7, UDG_8, UDG_13, UDG_23, Atlantic cod UDG or in the absence of enzyme (No UDG). *A*, reactions after a 10-min incubation at 25 °C with each UDG. *B*, reactions after a 10-min incubation at 25 °C with each UDG following a 10-min pre-incubation at 50 °C to promote enzyme inactivation. In both panels, complete degradation of the uracil-containing template shifts the signal to ∼10 cycles later (Ct ≈ 30), where only the 10^2^-copy thymine-only template is amplified. Full data in Figs. S4 and S5.

We next evaluated the baseline catalytic competence of the best-expressed UDGs using a qPCR-based template depletion assay that mirrors standard dUTP/UDG carryover prevention systems. Each reaction contained 10^5^ copies of a human PPIA template in which ∼60% of thymine residues were replaced with uracil, together with 10^2^ copies of a matched thymine-only template. This dual-template design distinguishes selective degradation of uracil-containing DNA from global qPCR failure, as complete depletion of the U-containing template still permits amplification of the thymine-only template. Given that a tenfold reduction in template concentration produces an increase of ∼3.3 Ct cycles, the 10^3^-fold difference between the U-containing and thymine-only templates (10^5^ vs 10^2^ copies) is expected to yield an ∼10-cycle Ct shift when the U-containing template is fully degraded. As shown in Figures 2*A* and S4, the majority of the tested UDGs efficiently suppressed amplification from the U-containing template, yielding Ct values consistent with near-complete reduction of the effective template concentration to the 10^2^-copy thymine-only background. At 25 °C, Atlantic cod UDG, a widely used thermolabile benchmark from a cold-adapted marine source, also fully degraded the U-containing template. In contrast, reactions containing UDG_13 or UDG_23 showed minimal Ct shifts relative to the no-UDG control, indicating low catalytic activity under the assay conditions (Fig. 2*A*).

To probe thermolability, we preincubated each enzyme for 10 min at 50 °C before conducting the template-depletion assay. As expected, Atlantic cod UDG was effectively inactivated under these conditions, resulting in amplification curves that overlapped with the no-UDG control (Figs. 2*B* and S5). Interestingly, only UDG_7, UDG_13, and UDG_23 exhibited a similar complete loss of activity following thermal pre-incubation. Since UDG_13 and UDG_23 already had reduced activity at 25 °C, their apparent thermosensitivity probably reflects inherently low catalytic efficiency rather than a genuine temperature-dependent switch (Fig. 2*A*). In contrast, UDG_7 maintained strong activity at 25 °C but fully lost function after the 50 °C pre-incubation, closely resembling the functional profile of Atlantic cod UDG. Two other UDGs (UDG_6 and UDG_24) showed partial inactivation (Fig. S5), while the remaining enzymes kept near-full activity after pre-incubation at 50 °C, resulting in approximately 10-cycle Ct shifts consistent with complete degradation of the uracil-containing template. Therefore, although most UDGs in this panel are active at 25 °C, only UDG_7 exhibits the ideal combination of high activity at low temperature and sharp inactivation at 50 °C required for molecular diagnostic applications. Although UDG_8 shares 82% sequence identity with UDG_7 (184 of 225 residues are identical) and is its only partner in clade A (Fig. S1), the conserved residues presumably support their similar catalytic performance, whereas differences at the remaining positions are apparently sufficient to render UDG_8 less thermolabile.

**Figure 3:**
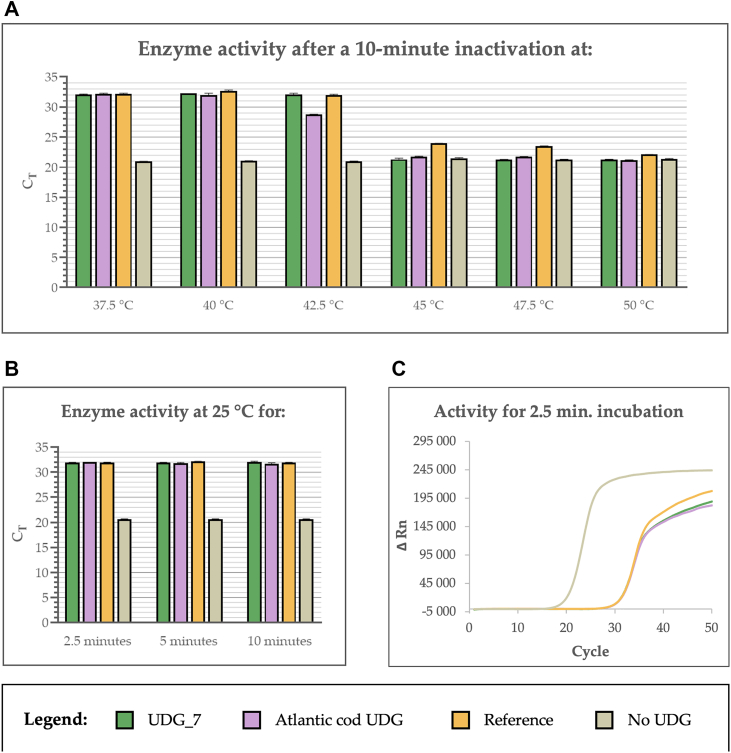
**Benchmarking UDG_7 against Atlantic cod UDG and a commercial thermolabile UDG.***A*, UDG_7, Atlantic cod UDG and a commercial thermolabile UDG (NEB M0372) were pre-incubated for 10 min at the indicated temperatures (37.5–50 °C) and then incubated at 25 °C with 10^5^ copies of a *C. albicans RPR1* template containing 60% dUTP plus 50 copies of a thymine-only template. Bars show mean Ct values after qPCR amplification. The “No UDG” control corresponds to reactions in which the template mixture was not exposed to UDG. *B*, the same three enzymes were incubated for 2.5, five5 or 10 min at 25 °C with 10^5^ copies of a *C. albicans RPR1* template containing 60% dUTP, supplemented with low-copy thymine-only templates. Bars show mean Ct values after qPCR amplification. “No UDG” denotes reactions lacking enzyme. *C*, representative amplification curves for the 2.5-min condition in *panel B*, illustrating that all three UDGs reach essentially maximal Ct shifts within this short incubation time. Error bars represent the standard deviation from replicates (n = 3).

We then benchmarked UDG_7 against Atlantic cod UDG and a commercial reference thermolabile UDG (NEB #M0372) (Figs. 3 and S6) across the 37.5 to 50 °C temperature range that is highly relevant for UDG inactivation. Each enzyme was pre-incubated for 10 min at 37.5, 40, 42.5, 45, or 50 °C and assayed using the qPCR-based depletion assay with a *Candida albicans* RPR1 template containing 10^5^ copies of 60% dUTP, supplemented with 50 copies of a thymine-only template (Fig. 3*A*). A no-UDG control was used in every run. Consistent with prior results, UDG_7 and Atlantic cod UDG displayed very similar inactivation profiles, retaining strong activity after mild pre-incubations but becoming almost fully inactivated as temperatures approached 45 °C (Fig. 3*A*). The commercial UDG followed a similar trend but consistently retained detectable residual activity at higher temperatures, indicating slightly greater thermal stability and a less sharply defined inactivation window (Fig. 3*A*).

Finally, we compared the kinetics of uracil-containing template depletion under diagnostic-like conditions using the same qPCR-based assay. UDG_7, Atlantic cod UDG, and the commercial UDG were incubated at 25 °C for 2.5, 5, or 10 min, using an assay setup similar to that described earlier. All three UDGs achieved near-maximal Ct shifts within 2.5 min, with no appreciable improvement at longer incubation times, indicating rapid and near-complete removal of U-containing templates on the timescale relevant to routine molecular diagnostics (Fig. 3, *B* and *C*). Together, these results identify UDG_7 as a naturally occurring UDG that uniquely combines robust low-temperature activity with a sharp and readily adjustable thermolability window, along with high recombinant yield in *E. coli*. These properties make UDG_7 an attractive scaffold for further structural analysis and for engineering next-generation thermolabile UDGs tailored to modern molecular diagnostic workflows.

### Crystal structure of UDG_7 and conservation of the family-I UNG fold

**Figure 4:**
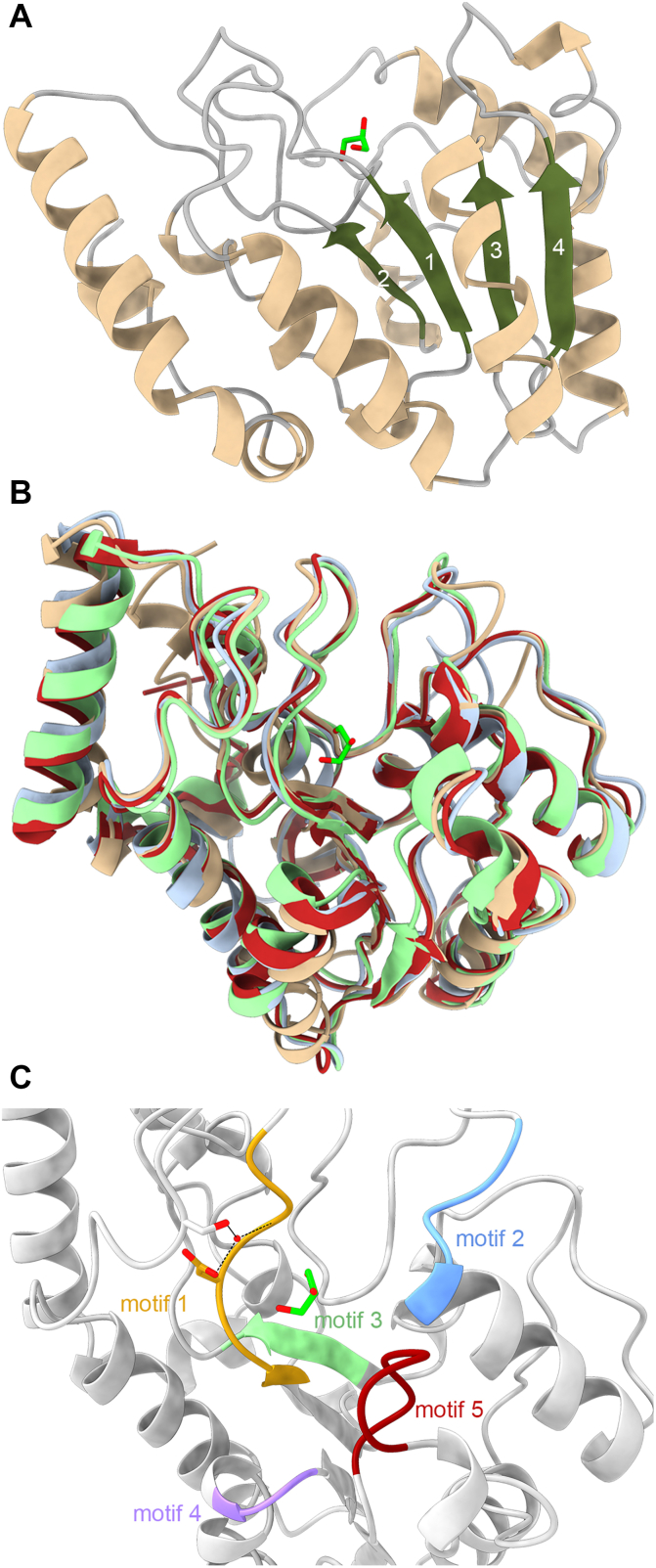
**Overall fold and core packing of UDG_7.***A*, cartoon representation of UDG_7 showing the canonical family-I UDG α/β fold, with the central four-stranded parallel β-sheet (*green*) flanked by α-helices (*beige*). The bound glycerol molecule in the active site is shown as *sticks*. *B*, superposition of UDG_7 (*beige*) with Atlantic cod UDG (*green*), human UNG (*red*) and *E. coli* UNG (*light blue*), highlighting the close overlap of the overall folds. *C*, close-up of the catalytic region of UDG_7, with the five conserved family-I motifs coloured as follows: motif 1 (water-activating loop, *yellow*), motif 2 (5′-compression loop, *blue*), motif 3 (uracil-binding pocket, *green*), motif 4 (3′-compression loop, *violet*) and motif 5 (Leu-intercalation loop, *red*). The catalytic Asp and His residues are shown as *sticks*.

To gain mechanistic insight into the thermolabile behavior of UDG_7, we determined its crystal structure (PDB 9TU4) and compared it with available representative UDG structures from psychrophilic and mesophilic organisms. The best-diffracting UDG_7 crystal belonged to the monoclinic space group C2, and the obtained dataset resulted in good-quality 2Fo−Fc maps, with clearly defined amino acid sidechains and ligands easily identifiable in the difference maps. No electron density was observed for the His tags. The best model was obtained at a 1.7 Å resolution. UDG_7 adopts the canonical family-I UDG architecture: a single-domain α/β fold consisting of a central four-stranded parallel β-sheet flanked by α-helices, forming a positively charged groove that cradles the DNA substrate (Fig. 4*A*). In the UDG_7 structure, a glycerol molecule from the cryoprotectant solution is observed in the uracil-binding pocket, occupying the central portion of the active-site cavity (Fig. 4*A*). Similar glycerol-bound, active-site configurations have been reported for *E. coli* UDG (PDB 3EUG) and Atlantic cod UDG (PDB 1OKB), where glycerol mimics key features of the uracil-binding environment without inducing major conformational changes, underscoring the shape complementarity and hydration properties of the UDG catalytic pocket (Fig. S7*A*).

A structural similarity analysis with PDBeFold/SSM using the obtained UDG_7 model identified close homologs from *Mycobacterium tuberculosis* (PDB 3A7N), *Deinococcus radiodurans* (PDB 2BOO), *Bacillus subtilis* (PDB 3ZOR), Atlantic cod (*Gadus morhua*, PDB 1OKB), human UNG (PDB 4SKN), and *E. coli* UNG (PDB 1LQJ). All these structures displayed Cα alignments with UDG_7 with RMSD values below 1.4 Å over at least 200 residues (Fig. 4*B*). Among these, only the Atlantic cod UDG has been characterized as a cold-adapted, highly thermolabile enzyme used to prevent PCR carryover contamination, whereas the human and *E. coli* UDGs are well-studied mesophilic counterparts. Superposition of UDG_7 with Atlantic cod, human, and *E. coli* UDGs reveals close alignment of all five conserved family-I motifs (Figs. 4*C* and S7*B*). The Asp-containing water-activating loop (motif 1), the Pro-rich 5′-compression loop (motif 2), the uracil-binding pocket (motif 3), the Gly–Ser 3′-compression loop (motif 4), and the Leu-intercalation loop (motif 5) are preserved in both geometry and relative positioning (Figs. 4*B* and S7*B*). As in other UDGs, the catalytic Asp in motif one and the His in motif five are precisely aligned for catalysis, consistent with the high specific activity observed for UDG_7. Most residues lining the uracil-binding pocket are also conserved across the four structures.

Active-site loop conformations further indicate that UDG_7 retains the structural machinery for the canonical base-flipping and glycosidic bond cleavage mechanism described for the human UNG-DNA complex (PDB 4SKN). The Leu intercalation loop inserts a bulky hydrophobic side chain (Leu191 of UDG_7, Leu272 in human UDG) into the minor groove of the DNA chain, while neighboring residues orient the His side chain to allow hydrogen-bonding to the uracil O_2_ and polarization of the scissile bond. The Gly–Ser loop clamps the 3′ phosphate in a geometry closely matching that of the deformed DNA backbone seen in human UDG substrate and product complexes. In contrast, a local variation is observed in the Pro-rich loop that anchors the 5′ phosphate, where UDG_7 contains an Arg at position 88 instead of the highly conserved Pro found in other structures. Despite this change, the side-chain position of the hydrogen-bonding Ser89 remains essentially unchanged, suggesting that the 5′ backbone clamp is preserved despite this sequence variation. Overall, these results show that UDG_7, like other family-I UDGs, depends on strain and stereoelectronic effects in the substrate rather than on unusual active-site chemistry to facilitate uracil flipping and catalysis.

Mapping the refined B-factors onto the UDG_7 surface shows that residues within the catalytic motifs are among the most rigid regions of the UDG structure, despite the enzyme's overall thermolabile behavior (Fig. S7, *C* and *D*). This observation supports the hypothesis that differences in thermal stability among UDGs do not originate from alterations in the catalytic machinery but instead from variations outside the active site. Notably, the most prominent differences between UDG_7 and Atlantic cod UDG, as well as their more thermostable human and *E. coli* counterparts, mainly lie in surface electrostatics. Atlantic cod UDG exhibits a more positively charged surface around the DNA-binding groove and fewer stabilizing salt bridges in the C-terminal half of the protein than human UDG. These features linked to increased catalytic efficiency at low temperatures and rapid thermal inactivation (17, 18, 19). In line with this cold-adapted profile, UDG_7 is notably arginine-rich, with 23 Arg residues, 19 of which are solvent-exposed. This represents a significant enrichment relative to the 9 to 11 Arg residues observed in the related enzymes studied here. Unexpectedly, UDG_7 also maintains a relatively high number of salt bridges, eight per monomer, similar to *E. coli* UDG and higher than in Atlantic cod UDG, which has four. This implies that the reduced thermostability of UDG_7 is not primarily due to the absolute number of ion pairs, but rather to their spatial arrangement and the dominance of solvent-exposed over deeply buried electrostatic interactions. In summary, the comparative structural analysis suggests that UDG_7 thermolability stems from the Arg-rich, highly charged surface stabilized by numerous solvent-exposed salt bridges. These non-catalytic features leave the active site essentially unchanged and offer clear opportunities to engineer additional thermal destabilization.

### Structure-guided saturation mutagenesis of non-catalytic determinants of UDG thermolability

Building on this framework, we next asked whether UDG_7 thermolability could be tuned in a controlled manner by modifying non-catalytic contacts while preserving the conserved catalytic core. This reasoning is consistent with previous work showing that subtle changes in local hydrogen-bond networks or helix-capping motifs can trigger large shifts in protein stability while leaving the overall fold and catalytic machinery essentially intact (28). We therefore hypothesized that, within family-I UDGs, overall stability is predominantly encoded by surface and internal regions that modulate electrostatics, packing, and conformational flexibility around a conserved active site, and that these determinants can be systematically reprogrammed without abolishing catalytic function.

**Figure 5:**
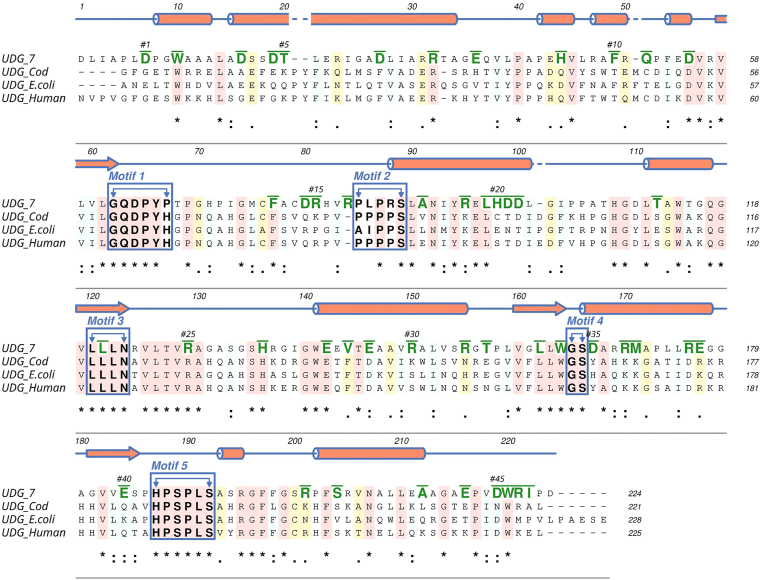
**Structure-guided identification of non-catalytic positions in UDG_7 for single-site saturation mutagenesis.** Multiple-sequence alignment of UDG_7 with Atlantic cod UDG, *E. coli* UNG, and human UNG. The secondary-structure of UDG_7 (α-helices and β-strands) is shown above the alignment. Blue boxes delineate the five conserved family-I motifs (1, 2, 3, 4, 5), and their consensus sequences are indicated below. Residues chosen for diversification in the single-site variant library (SSVL) are highlighted in *green* and numbered according to their SSVL position; sites were selected based on structural context (solvent-exposed polar/charged residues, surface hydrophobics, and buried core residues contributing to local packing or electrostatic networks). Except for Leu24 in motif three and Leu33 and Trp34 in motif 4, all diversified positions lie outside the catalytic motifs. These three residues were included only where structural modelling indicated that side-chain substitutions could be accommodated without perturbing the geometry required for base flipping and catalysis. Phe77 (SSVL position 13) contributes directly to the substrate-binding pocket, where its aromatic side chain forms π-stacking interactions with the uracil base.

To test this hypothesis in a structurally grounded and systematic manner, we designed a single-site variant library (SSVL) on the UDG_7 scaffold, targeting regions outside the canonical catalytic motifs. A total of 48 positions were selected based on three structural criteria and are annotated in detail in Figure 5 and Table S3. First, we prioritized solvent-exposed or shallowly buried polar and charged residues that participate in local hydrogen-bond networks, helix N-caps, or salt-bridge “electrostatic clamps” (e.g., D7, D27, E36, T112, E142, E146, R150, R176, E184, R201, D219, and R221). Substitutions at these nodes are expected to modulate local flexibility and long-range electrostatic coupling without directly perturbing the uracil-binding pocket. Second, we included several surface or near-surface hydrophobic residues that contribute to shallow packing layers (such as A91, V144, L162, W164, and A212), where replacement by more polar or differently sized side chains is predicted to weaken non-polar clustering and subtly disrupt packing. Third, we targeted a limited set of deeply buried hydrophobic “core packing nodes” and steric buttresses (for example, W10, F49, L97, L121, and I222) located away from the conserved catalytic motifs, to probe how modest perturbations of the interior scaffold influence global stability. For all selected sites, inspection of the UDG_7 structure indicated that side-chain diversification could be accommodated without grossly distorting the conserved family-I motifs or the geometry required for base flipping and catalysis, while still allowing systematic perturbation of the surrounding non-catalytic stability network. Candidate sites were cross-referenced against UDG_7-specific structural features and sequence/structural alignments with Atlantic cod, human and *E. coli* UDGs, with preference given to positions where UDG_7 harbors unique, atypically bulky or charged side chains (Fig. 5). Two selected positions lie within the conserved family-I motifs: Phe77 (SSVL position 13) provides the aromatic stacking platform for the uracil base in the substrate-binding pocket, whereas Leu121 (SSVL position 24) resides in uracil-binding motif three but is buried and spatially remote from the pocket (Fig. 5, Table S3). Substitutions at these sites were therefore expected to modulate local packing or fine-tune base-stacking interactions, eventually affecting the geometry required for base flipping and catalysis.

For each of the 48 selected positions, we constructed a focused single-site saturation series in which the wild-type residue was replaced by each of the other 19 amino acids. All variants were synthesized in a pooled gene-synthesis reaction such that each targeted position was represented by a sub-library containing the 19 alternative codons within an otherwise identical UDG_7 coding sequence (Table S4). This design yields a theoretical library of 912 single-amino-acid variants (48 × 19), each bearing a unique substitution at a defined position and no additional mutations. The diversified UDG_7 fragments were cloned into the pHTP1 expression vector, generating a plasmid library suitable for functional screening and deep mutational profiling. Next-generation sequencing (NGS) of the cloned library confirmed broad representation of the designed variants but revealed that three adjacent sites (positions 37, 38 and 39) were under-represented or essentially absent and were therefore excluded from downstream analyses (Table S4). For the remaining 45 positions, the majority of the 19 possible amino-acid substitutions were detected, with individual variants quantified as a percentage of total reads per position (Table S4). After quality filtering, the final library comprised up to 855 distinct UDG_7 variants, providing near-saturation coverage of a structurally defined set of non-catalytic sites and enabling systematic interrogation of thermolability determinants.

### Thermal profiling of pooled single-site variants reveals non-catalytic thermolability hotspots

To translate the structure-guided SSVL into functional information on stability, we next asked which positions in UDG_7 tolerate extensive side-chain diversification while preserving solubility, and which positions are intrinsically predisposed to yield thermolabile variants. For each SSVL position, we expressed and purified the pooled set of 19 single-amino-acid variants, together with wild-type UDG_7, and monitored thermal unfolding using fluorescence-based melting assays. Because each sample contains a mixture of variants differing only at a single position, the apparent melting temperature (Tm) of the pool reports on the underlying distribution of individual Tm values at that site. Thus, positions where most substitutions are destabilizing are expected to shift the pool Tm below that of UDG_7, whereas positions dominated by neutral or stabilizing substitutions should yield Tm values similar to or higher than the wild-type reference.

Out of the 45 positions covering all required variants (Table S4), expression screening showed that pooled variants at 16 sites predominantly produced insoluble protein across multiple expression conditions, in striking contrast to UDG_7 and most other pools (Table S3). These solubility-sensitive sites include a set of buried aromatic and hydrophobic core residues (Phe49, Leu121, Leu162, Trp164, Trp220, and Ile222) along with surface or partially buried scaffolding and electrostatic nodes (Asp7, Trp10, Asp80, Arg81, Asp99, Asp100, Arg129, His136, Glu142, and Arg155) that structural analysis previously identified as core packing hubs, backbone supports, or key participants in salt-bridge or hydrogen-bond networks in UDG_7 (Table S3). The widespread loss of solubility observed with saturation mutagenesis at these sites suggests they are part of a highly constrained structural framework where most amino acid substitutions are incompatible with proper folding, leading to misfolding or aggregation rather than targeted thermal stability adjustments. As a result, these 16 positions were excluded from further biophysical and functional analyses. The remaining 29 positions, for which pooled libraries yielded robust amounts of soluble protein while retaining full codon diversity, were carried forward for detailed thermal profiling.

**Figure 6:**
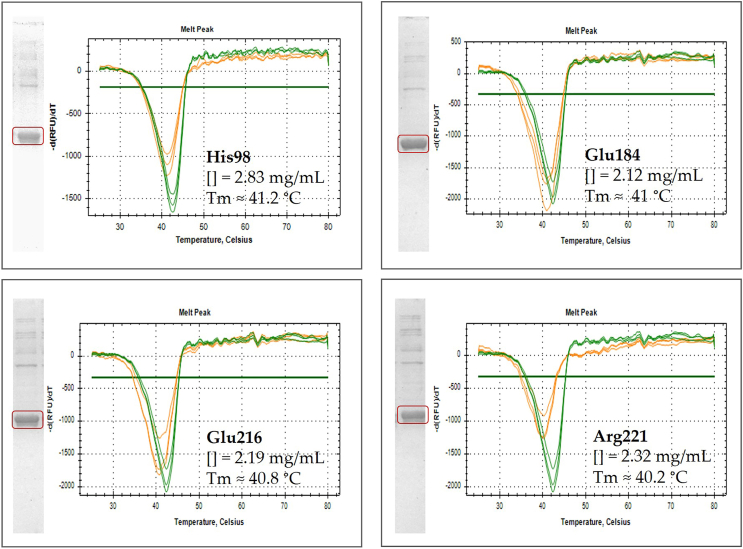
**Thermal profiling of pooled single-site variant libraries identifies thermolability hotspots in UDG_7.** Derivative melting curves (−dRFU/dT *versus* temperature) for wild-type UDG_7 (*green*) and pooled single-site variant libraries (*orange*) at representative positions. Each pool contains the 19 possible amino-acid substitutions at a single site. Panels illustrate positions classified as thermolability hotspots, in which the pooled variants exhibit a reproducible decrease in apparent melting temperature (Tm) relative to wild-type UDG_7 while remaining soluble. Protein concentration ([]) and apparent Tm values (Tm) for each pool are indicated. SDS-PAGE panels to the *left* of each plot confirm the integrity and solubility of the pooled samples. Full data is presented in Figs. S7 and compiled in Table S3.

For these 29 SSVL positions, pooled UDG_7 variants were recovered at yields comparable to the wild type, enabling quantitative analysis of their thermal unfolding profiles (Figs. 6 and S8; Table S3). Across these positions, we observed a spectrum of unfolding behaviors. Some pools yielded sharp, single cooperative transitions with well-defined derivative maxima, whereas others showed broadened or shallow peaks, consistent with mixtures of variants spanning a range of stabilities. For a small subset of pools, no clear transition could be resolved, and a reliable midpoint could not be assigned (classified as “ND” in Table S3). Given this intrinsic heterogeneity, we interpreted the apparent Tm of each pool not as an absolute thermodynamic parameter for individual variants, but as an effective, position-specific readout of the balance between stabilizing and destabilizing substitutions tolerated at that site.

Using UDG_7 (Tm ≈ 42.5 °C) as an internal reference, we then focused on positions that met three criteria simultaneously: (i) robust soluble expression of the pooled library, (ii) derivative traces with at least discernible cooperative transitions (classified as Low to Excellent peak definition), and (iii) reproducible shifts of the main unfolding transition to lower temperature relative to UDG_7. Sixteen positions met these criteria and were identified as candidate thermolability hotspots in UDG_7: Asp19, Asp27, Gln51, Asp55, Arg84, Ala91, His98, Thr112, Arg150, Asp167, Glu184, Arg201, Ala212, Glu216, Asp219, and Arg221 (UDG_7 numbering; Table S3). For all these sites, pooled variant Tm values ranged from approximately 39 to 41.8 °C, indicating consistent yet moderate destabilization compared to the wild type while maintaining cooperative unfolding behavior.

Structural annotation of these 16 sites reveals that they form a non-catalytic stability network spanning the UDG_7 scaffold (Fig. 5, Table S3). Several hotspots coincide with, or are near, electrostatic clamps, polar hubs, and other structured polar interaction nodes that often connect surface segments to the core or to the C-terminal tail (Asp27, Gln51, Asp55, His98, Thr112, Arg150, Glu184, Arg201, Asp219, and Arg221). This indicates that disrupting long-range salt-bridge and hydrogen-bond networks can effectively adjust overall stability without directly affecting the uracil-binding pocket. Other sites act as local structural supports on the surface or within shallow packing layers, including surface scaffold residues (Asp19, Arg84, Asp167, and Glu216) and core-packing nodes (Ala91 and Ala212), where minor side-chain changes are likely to influence packing density or short-range hydrogen bonding (Table S3). Collectively, these findings suggest that the chosen hotspots are located at positions where a single side chain’s identity exerts a disproportionately strong influence on the overall stability of UDG_7, while remaining compatible with proper folding and solubility when mutated. On this basis, we defined these 16 positions as thermolability hotspots in UDG_7 and used them as the principal entry points for isolating individual thermolabile variants that preserve catalytic function, while acknowledging that additional SSVL sites could yield useful destabilizing substitutions when interrogated at single-variant resolution.

### High-throughput screening of hotspot positions identifies thermolabile, catalytically competent UDG_7 variants

Having defined 16 non-catalytic thermolability hotspots through pooled thermal profiling, we next sought to resolve the individual sequence determinants responsible for this behavior. For each hotspot position, the corresponding SSVL pDNA pools were transformed into *E. coli* BL21 and 30 independent colonies were expressed in auto-induction medium, yielding a panel of 480 crude extracts (16 positions × 30 clones) encompassing single-amino-acid variants of UDG_7. Cell lysates were prepared by chemical lysis without DNase I to preserve plasmid DNA, and each extract was subjected to a three-step functional screen designed to capture the desired diagnostic profile: robust UDG activity at 25 °C, followed by efficient inactivation at 35 °C and 45 °C. Representative plate layouts are shown in Fig. S9, and the full scoring is summarized in Table S5. This screen identified 114 of the 480 clones (≈24%) that exhibited the targeted “on–off–off” phenotype, remaining active at 25 °C while losing detectable activity at both 35 °C and 45 °C (Fig. S9, Table S5). Because most of these hits map to thermolability hotspots, they reinforce the hypothesis that these sites are particularly sensitive levers for tuning UDG_7's thermal stability.

Plasmid DNA corresponding to these 114 “on–off–off” clones (active at 25 °C and inactive at 35 °C and 45 °C; Fig. S9, Table S5) was recovered by re-transformation into *E. coli* DH5α. Despite an expected loss of material due to endonuclease-mediated degradation during extract preparation, 66 plasmids were successfully recovered and sequenced (Table S6). Sequence analysis revealed 54 unique single-amino-acid substitutions that confer the desired functional profile, distributed over 20 distinct positions in the UDG_7 scaffold (SSVL positions 4–6, 11, 12, 16, 17, 20, 21, 23, 28, 30, 35, 36, 40, 41, 43–45, and 47; Table S6). Seven substitutions mapped to positions that were not initially classified as thermolability hotspots in the pooled melting analysis (positions 5, 21, 28, and 36). This likely reflects a combination of low-level cross-contamination between SSVL libraries and the presence, at some non-hotspot sites, of minority thermolabile variants that do not strongly influence the pooled melting profile. Because these substitutions met the functional selection criteria robustly, the corresponding variants were retained for subsequent analyses.

All 54 UDG_7 variants were then expressed and individually purified to near homogeneity (Fig. S10), and their unfolding profiles were determined under the same conditions used for wild-type UDG_7 (T_m_ ≈ 42.5 °C; Fig. S11, Table S6). With few exceptions, the substitutions lowered the apparent melting midpoint, yielding T_m_ values clustered around ∼40 °C, with several variants reaching ∼36 to 38 °C. For a small subset, cooperative unfolding transitions could not be reliably defined and are reported as “ND” in Table S6. These data confirm that the crude-extract screen efficiently enriched for substitutions that further destabilize UDG_7 while preserving catalytic activity at low temperature.

**Figure 7:**
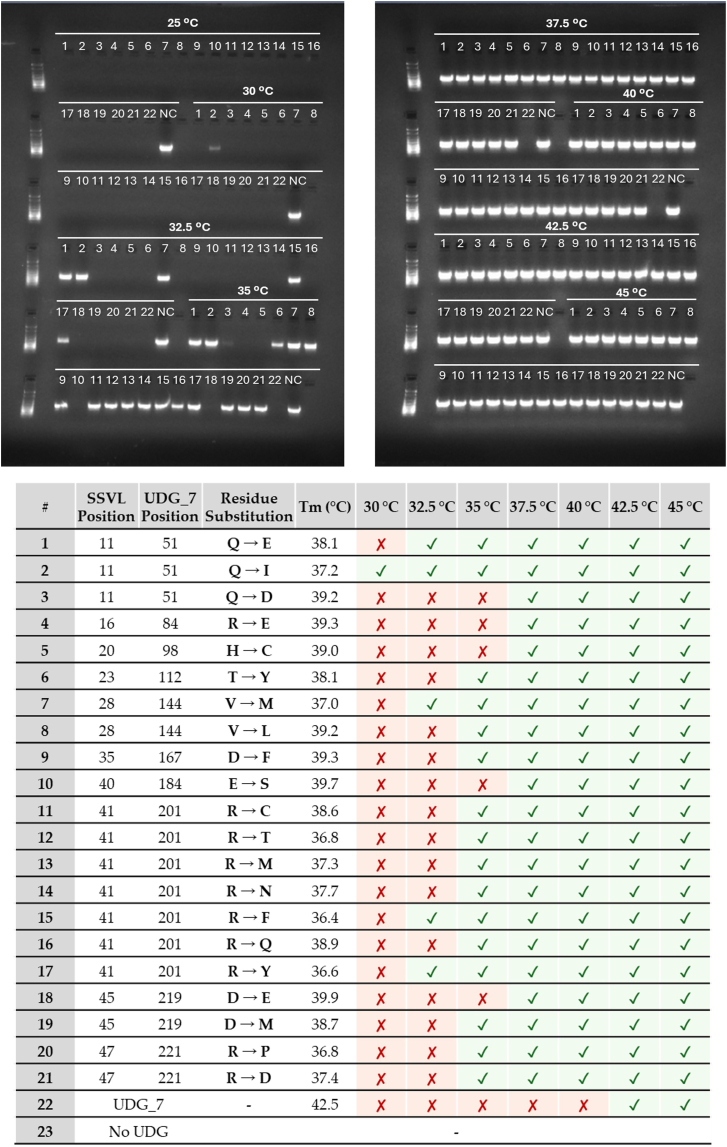
**Temperature-dependent activity profiles of the selected 21 thermolabile UDG_7 variants.***Top panels*: Agarose-gel–based activity and thermostability assays for wild-type UDG_7 (lane 22) and 21 selected thermolabile variants (lanes 1–21). Lane “NC” corresponds to the no-UDG control. UDG activity was assessed at 25 °C and tested for inactivation at temperatures between 30 °C and 45 °C by monitoring degradation of uracil-containing primers (see Experimental Procedures). Disappearance of the DNA band indicates active UDG; intact DNA indicates loss of activity. *Bottom panels*: Summary of temperature-dependent inactivation for the same variants. For each residue substitution (SSVL Position, UDG_7 Position, and mutation), the experimentally determined Tm and qualitative activity at each incubation temperature are shown (✔, thermal inactivation successful; ✘, no inactivation). Wild-type UDG_7 remains active over a broad temperature range, whereas the engineered variants display a narrow window of inactivation centered at *low* temperatures, losing activity sharply between ∼37.5 °C and 42.5 °C.

From this collection, we selected 21 variants originating from 10 different positions that combined clear melting transitions with the largest decreases in T_m_ (all with T_m_ < 40 °C; Table S6) for detailed biochemical characterization. Steady-state activity measurements and thermal-decay assays confirmed that all selected variants retain near-native UDG activity at 25 °C but, unlike UDG_7, undergo accelerated loss of function upon incubation at 30 to 37.5 °C (Fig. 7). Inspection of the activity matrix revealed that substitutions at positions 51, 112, 144, 167, 201, 219, and 221 reproducibly shifted the inactivation window in at least one clone into the 30 to 35 °C range, whereas variants at positions 84, 98, and 184 were only inactivated at ≥ 37.5 °C, thus more similar to wild-type UDG_7 (Fig. 7, Table S6). Consistent with these patterns, the most favorable profiles were observed for Q51I, T112Y, V144M, D167F, and D219M, two replacements at position 221 (R221P and R221D), and several substitutions at position 201 (notably R201F and R201Y), all of which display T_m_ values of ∼36 to 39.9 °C and lose detectable activity between 30 °C to 35 °C while remaining fully active at 25 °C (Fig. 7, Table S5). On this basis, Q51I, T112Y, V144M, D167F, R201F, R201Y, D219M, R221P and R221D were identified as the most thermolabile yet catalytically competent UDG_7 variants (Fig. 7). Among all positions tested, Arg201 (SSVL position 41) was the most consistently productive entry point for engineering thermolability while preserving catalytic competence: multiple substitutions at this site shifted the inactivation boundary downward, and R201F and R201Y in particular combined full activity at 25 °C with early inactivation and a pronounced reduction in thermal stability (Tm ≈ 36.4–36.6 °C *versus* 42.5 °C for UDG_7; Fig. 7, Table S6).

## Discussion

Building on an approach that combines evolutionary sequence mining, structural analysis, and targeted saturation mutagenesis, we systematically define the non-catalytic determinants that encode UDG thermolability in a diagnostic context. Across the UDG_7 scaffold, thermolability can be tuned over a broad operational range (Tm ≈ 36–45 °C), producing corresponding, position-dependent shifts in functional inactivation while preserving robust catalytic activity at 25 °C. From a phylogenetically diverse 24-member panel, UDG_7 emerged as a naturally thermolabile enzyme that couples strong low-temperature activity with sharp inactivation near 45 °C, closely mirroring benchmark Atlantic cod UDG. The crystal structure of UDG_7 confirms that the five family-I motifs and the catalytic Asp/His pair are essentially similar to those of human, *E. coli*, and cUNG (19, 26, 31, 32, 33). Together with prior analyses of cold-adapted UDGs (17, 18, 19), these findings support a model in which diagnostically useful thermolability is encoded primarily by distributed, non-catalytic interactions in the scaffold rather than by alterations in the conserved active-site architecture.

The structure-guided single-site variant library provides a quantitative dissection of the non-catalytic stability layer and shows that it offers the most tractable route to adjust UDG thermolability without eroding catalytic competence. In practice, saturation mutagenesis partitioned the UDG_7 scaffold into two distinct classes of sites with sharply different outcomes. A first class includes positions that act as folding gatekeepers, where diversification largely reduces solubility, indicating that these residues are part of a rigid, highly constrained core structure. These include deeply buried packing nodes enriched in aromatics and hydrophobics (Trp10, Phe49, Leu121, Leu162, Trp164, Trp220, and Ile222), along with partially buried polar hubs and electrostatic linkers that connect secondary-structure elements into the interior scaffold (Asp7, Asp80, Arg81, Asp99, Asp100, Arg129, His136, Glu142, and Arg155). Notably, many of these “insoluble” positions are involved in dense backbone scaffolding and long-range polar interactions, including helix-capping interactions, salt bridges, and hydrogen-bond clusters that connect distant segments. Disruption of this network usually causes misfolding rather than a gradual loss of stability.

In contrast, a second class comprises sites that tolerate extensive substitution while remaining soluble and folded, and are thus reported as thermolability-sensitive levers, shifting the pooled melting transition below that of wild-type UDG_7. These hotspots are mainly surface-exposed or near-surface residues that exist in relatively localized electrostatic or hydrogen-bond networks, such as Asp19, Asp27, Gln51, Asp55, Arg84, His98, Thr112, Arg150, Asp167, Glu184, Arg201, Glu216, Asp219, and Arg221. They also include two shallow packing nodes, Ala91 and Ala212, which are partially buried but remain close to solvent-exposed surfaces. Importantly, these positions are not part of tightly packed regions that fail under saturation mutagenesis. Instead, they decorate the surface and weakly connect to the stability network. Substitutions at these sites tend to weaken local electrostatic clamps, helix-cap hydrogen bonds, or shallow packing contacts, thereby lowering Tm while still maintaining solubility and activity. This behavior aligns with classical temperature-sensitive design principles, in which local hydrogen-bond and salt-bridge microcircuits, together with helix caps and shallow packing features, exert disproportionate control over thermal robustness, whereas perturbations of deeply buried core nodes tend to be catastrophic rather than tunable (26, 27, 28). In that sense, the UDG_7 map also clarifies why purely depth-based predictors that prioritize buried hydrophobics as Ts candidates are not, on their own, optimal for engineering diagnostically useful thermolability, because the most deeply buried nodes in UDG_7 primarily enforce fold integrity rather than offering adjustable inactivation windows (29).

**Figure 8:**
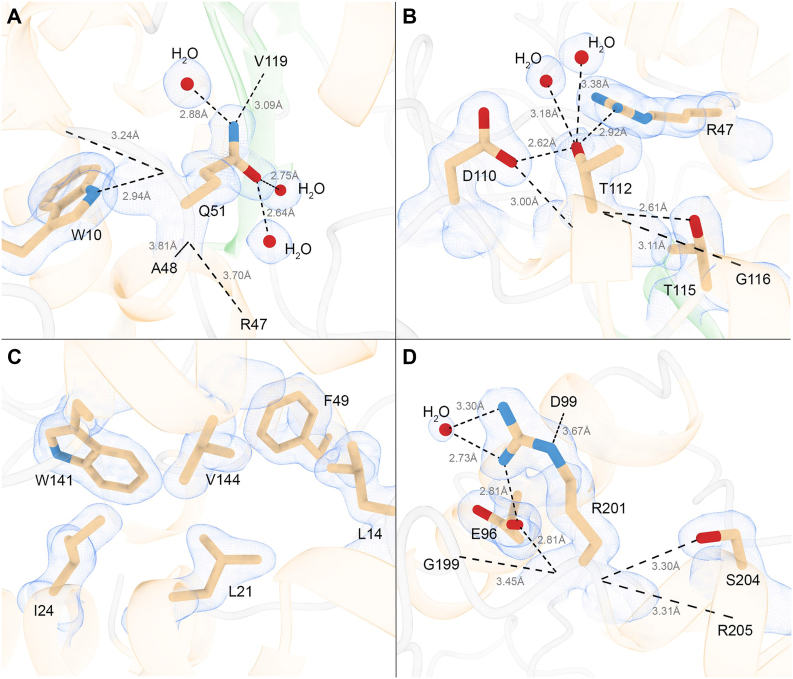
**Local interaction environments at key thermolability-sensitive positions in UDG_7.***A–D*, close-up views of the structural contexts of Gln51 (*A*), Thr112 (*B*), Val144 (*C*), and Arg201 (*D*). UDG_7 is shown as a cartoon representation (helices in *brown*, β-strands in *green*, loops in *gray*). Side chains of residues contributing to the local interaction environment are shown as *sticks*. Water molecules are shown as *red spheres*. *Dashed lines* indicate polar contacts (hydrogen bonds and, where applicable, salt-bridge interactions), with distances shown in Å. *Panels* (*A*, *B*, and *D*) highlight organized polar microcircuits around Gln51, Thr112, and Arg201 that are consistent with their identification as thermolability-sensitive nodes. *Panel C* shows the local environment surrounding Val144, which lies within a shallow hydrophobic pocket formed by nearby residues (including Ile24, Leu21, Leu14, Phe49, and Trp141).

High-throughput functional screening of the hotspot set yielded 54 single-site variants with the desired “on–off–off” behavior and distilled a small group of substitutions that best combine catalytic competence with an earlier loss-of-function window. In purified-enzyme assays, Q51I, T112Y, V144M, D167F, R201F, R201Y, D219M, R221P, and R221D retained near-native activity at 25 °C yet inactivated more readily than UDG_7 upon mild thermal challenge (Fig. 7). Importantly, the inactivation boundary was position- and substitution-dependent: Q51I, V144M, and the strongest Arg201 variants (R201F, R201Y) were already inactive by 30 to 32.5 °C, whereas T112Y, D167F, D219M, R221P, and R221D remained active through 32.5 °C but switched off at 35 °C (Fig. 7). Across the full set, Arg201 (SSVL position 41) emerged as the most consistently productive entry point for engineering thermolability while preserving activity, with multiple substitutions shifting the functional boundary downward and the two standout variants, R201F and R201Y, delivering a pronounced reduction in thermal stability (Tm ≈ 36.4–36.6 °C *versus* 42.5 °C for UDG_7) alongside early inactivation. Structurally, these substitutions map to previously annotated non-catalytic nodes, including polar-network and electrostatic-clamp positions (Q51, T112, R201, D219, and R221) and backbone-scaffold sites (V144 and D167). Consistent with their assigned contact patterns, the largest functional shifts are associated with perturbations of structured surface or interface interactions (Fig. 8), whereas the V144 and D167 effects are more readily rationalized by altered local architecture and surface backbone-supported constraints. Together, these results sharpen the hotspot model into a practical design rule where a modest disruption of a compact, noncatalytic stability network can shift the operational “UDG-off” window downward while leaving the conserved catalytic core functionally intact.

In summary, UDG thermolability can be tuned by weakening a surface stability network without disrupting the conserved catalytic architecture. This property is particularly important for molecular diagnostic formats that begin amplification at moderate temperatures and therefore cannot rely on an early high-temperature step to eliminate UDG activity. Under these conditions, incomplete shutdown often forces conservative UDG dosing, thus limiting carryover protection. The variants described here shift the thermal inactivation boundary downward while preserving low-temperature activity, providing a practical route to improve carryover control without compromising assay sensitivity.

## Experimental procedures

### Selection of a representative UDG panel from a large sequence compendium

A total of 8482 uracil-DNA glycosylase (UDG) sequences from psychrophilic, low-temperature-adapted mesophilic, or mesophilic bacteria were compiled from publicly available databases and curated to retain only the UDG domain of the different enzymes. Sequences lacking the conserved motif 1 (GQDPY) characteristic of family 1 UDGs, displaying extensive N- or C-terminal truncations, or containing ambiguous residues (B, J, O, U, X, and Z) were excluded. To minimize redundancy and avoid over-representation of closely related strains, the curated set was clustered using MMSeqs2 from the Galaxy Project (https://usegalaxy.eu) applying a 90% identity threshold and 80% coverage on the Easy-cluster workflow (35), and a single representative sequence was retained from each cluster. The resulting 2775 non-redundant representatives were aligned with MAFFT, the sequence alignment was curated with TrimAI and a maximum-likelihood phylogeny was then inferred with FastTree, all within the “A La Carte” workflow of NGPhylogeny.fr (36). The unrooted phylogenetic tree was annotated with phylum taxonomic ranks, ecological niches and thermal preferences of the source organisms and visualized using TreeViewer (37). We defined a panel of 24 enzymes that together maximize coverage of deep clades, taxonomic breadth, and ecological niches, with an intentional enrichment for cold-adapted lineages likely to encode thermolabile UDGs. For clarity throughout the manuscript, these enzymes are referred to as UDG_1 to UDG_24, and their source organisms, accession numbers, ecological origins, and growth temperature ranges are summarized in Table S1.

### Strains, plasmids, molecular cloning, expression, and purification of recombinant enzymes

*E. coli* strains used for gene cloning (DH5α) and expression (BL21(DE3)) were provided by NZYtech. For DNA extraction and purification, the NZYMiniprep kit (NZYtech) was used. The genes encoding the enzymes selected were synthesized using a gene synthesis service provided by IDTDNA and cloned into the prokaryotic pHTP1 expression vector, which contains two poly-histidine(6xHis) sequences (N- and C-terminal) that allowed subsequent recombinant protein purification by immobilized metal ion affinity chromatography (IMAC). Molecular cloning was performed using the Ligation-Independent Cloning (LIC) method with the NZYEasy Cloning & Expression kit I (NZYtech). Positive clones were analyzed by Sanger DNA sequencing through the Macrogen Europe CES service (Amsterdam, The Netherlands) and using specific primers (Primer Forward: 5′-GCGAAATTAATACGACTCACTATAGGGG-3′; Primer Reverse: 5′-GGTTATGCTAGTTATTGCTCAGCG-3′) to confirm the correct insertion and integrity of the sequence. The recombinant enzymes were expressed in *E. coli* BL21(DE3) grown in 250 ml of NZY Auto-Induction LB medium (NZYtech), cells were collected through centrifugation, chemically lysed in NZY Bacterial Cell Lysis Buffer (NZYtech) supplemented with 2 μl of lysozyme and 2 μl of DNase I per 1 ml of buffer as recommended by the manufacturer, and purified through IMAC resorting to an ÄKTA go protein purification system (Cytiva) according to the following protocol: first, the column was equilibrated with 25 ml of buffer A (50 mM Na_2_HPO_4_, 10 mM Imidazole, 1 M NaCl, pH 7.5); second, the protein extract was fully loaded into the column; third, the column was washed with 150 ml of buffer A; fourth, the column was washed with 550 ml of buffer A supplemented with 1% Tween20; fifth, the column was once again washed with 100 ml of buffer A; and finally protein was eluted in 2.5 ml fractions using buffer E (50 mM Na_2_HPO_4_, 300 mM Imidazole, 1 M NaCl, pH 7.5). After IMAC purification, proteins were stored at 4 °C in buffer E. The proteins’ relative solubility, expression levels, and estimated molecular weight were confirmed by SDS-PAGE. Gels were imaged using a Gel Doc XR + System (Bio-Rad). For protein electrophoresis, standard 14% SDS gels were prepared in-house using NZYtech’s acrylamide/bis-acrylamide.

### Analysis of protein sequences, structure prediction and phylogeny estimation

The theoretical molecular weight (MW), molecular extinction coefficient (ε) and isoelectric point (pI) of the 26 selected protein sequences (including Atlantic cod UDG, *E. coli* UDG and 24 psychrophilic variants) were estimated using the ProtParam tool (38) (ExPASy, SIB, Lausanne, Switzerland: https://web.expasy.org/protparam/). The Clustal Omega EBI tool (39) (European Bioinformatics Institute, Hinxton, United Kingdom: https://www.ebi.ac.uk/jdispatcher/msa/clustalo) was used to align the selected protein sequences. Phylogenetic analysis was conducted based on the amino acid sequences of the 19 proteins using the “One Click Mode” of NGPhylogeny.fr (36) (Institut Pasteur, Paris, France: https://ngphylogeny.fr/workflows/oneclick/). Protein secondary structure prediction was performed using JPred four online tool (University of Dundee: http://www.compbio.dundee.ac.uk/jpred/) in conjunction with Jalview software (40) (Version 2.11.4.1).

### Thermal shift assay

The unfolding temperatures of the proteins analyzed were estimated using a thermal shift assay (36) with SYPRO Orange Protein Gel Stain (Thermo Fisher Scientific). The following reaction mix was prepared for each protein: 4x SYPRO Orange Protein Gel Stain, 1.25 μg protein and water up to 25 μl. For each protein, this reaction was prepared in quadruplicate, and the reaction mixes were distributed on a 96 Well Semi Skirted PCR plate (NZYtech), which was then sealed with a qPCR adhesive clear plate seal (NZYtech). The plate was then placed in a CFX Opus 96 Real-Time PCR System (Bio-Rad), and the following melting curve protocol was used: a temperature increase of 0.2 °C from 25 °C to 65 °C, with fluorescence recorded every 15 s. Fluorescence emission was measured using the FRET channel.

### Master mixes, templates, primers and probes

A qPCR probe master mix (2×) was prepared as follows: 2× reaction buffer for NZYTaq DNA polymerase 10× (NZYtech), 5 mM MgCl_2_ (Sigma-Aldrich), 0.5 g/L BSA (Sigma-Aldrich), 4.5 μg mL^–1^ NZYTaq DNA polymerase (NZYtech), 54 μg mL^–1^ monoclonal mouse anti-Taq polymerase antibody (100 mg, YO Proteins AB, Sweden), 0.02% Tween-20 (Sigma-Aldrich), and water. A 100 mM dNTPs mix with a 3:2 ratio of uracil to thymine (referred to below as 60% U-dNTPs mix) was prepared by combining: 15 mM dUTP solution, 10 mM dTTP solution, 25 mM dATP solution, 25 mM dGTP solution, and 25 mM dCTP solution, all from Thermo-Fisher. The template DNAs used in these assays were synthetic DNA fragments obtained from IDTDNA, quantified using a NanoDrop One spectrophotometer (Thermo-Fisher), and diluted in IDTE 1x TE solution pH 8.0 (IDTDNA) to obtain serial DNA concentrations ranging from 10^7^ to 10 copies/μl. The templates used in these assays included the Peptidylprolyl Isomerase A (PPIA) gene from *Homo sapiens* (NC_000007.14: 44795960-44803117) and the RNase P RNA 1 (RPR1) gene from *C. albicans* (DQ660433.1). The primers and probes for each target were ordered from IDTDNA, with their sequences listed in Table S7. For each target, 10× primer-probe mixes were prepared as follows: 4 μM forward primer, 4 μM reverse primer, 1.25 μM probe, and water to a final volume of 800 μl.

### Assessment of enzyme activity and thermostability by real-time PCR assays

The UDGs recombinantly produced were characterized regarding their activity and thermostability following established protocols in a real-time PCR (qPCR) setup for different targets and utilizing StepOnePlus Real-Time PCR System (Thermo-Fisher). Enzyme activity was evaluated by incubating DNA templates with each UDG for 10 min at 25 °C for the qPCR assay. Thermal inactivation was assessed by incubating DNA templates with each UDG (previously incubated at 35 or 45 °C) for 10 min at 25 °C before the qPCR reaction. All template incubations with UDG were performed by combining 5 μl of each enzyme (5 μg mL^–1^) with 10 μl of DNA. A typical qPCR reaction comprised of: 1x qPCR Probe Master Mix 2×, 0.2 mM 60% U-dNTPs mix (100 mM), 1× Primer + Probe Mix 10×, 1 μl of template DNA (previously incubated with active or thermally inactivated UDG) and water up to 20 μl. A commercial thermolabile UDG—Antarctic Thermolabile UDG from New England Biolabs—was used as reference for these assays.

### Crystallization of UDG_7

IMAC purified UDG_7 was buffer exchanged into 50 mM HEPES, pH 7.5 and further purified by size-exclusion chromatography using a HiLoad 16/60 Superdex 75 (Cytiva) in the Akta-go system, without removal of the 6xHis tags. The obtained fractions were pooled, buffer exchanged into water and concentrated to 18 mg mL^–1^ using an Amicon Ultra-15 centrifugal filter with a 10-kDa cutoff membrane (Millipore). Several crystallization conditions were tested by using the sitting-drop vapor-diffusion method, with the aid of an Oryx8 robotic nanodrop dispensing system (Douglas Instruments). The commercial kits JCSG+, Crystal Screen, and PEG/Ion (Hampton Research), and 80 in-house-prepared factorial solutions were used for the screening. Drops containing 1 μl of 18 mg⋅mL^–1^ UDG_7, mixed with 1 μl of reservoir solution, were set up at room temperature. The resulting plates were then stored at 20 °C. Crystal formation was observed under only one condition after approximately 30 days from the time the plates were set up. The successful condition was further optimized to produce high-quality crystals suitable for structural analysis. A total of four crystals were cryoprotected in well solution with 25% glycerol and flash-cooled in liquid N_2_ for data collection. Preliminary in-house X-ray diffraction experiments (microfocus IμS Bruker D8 Venture CuKα diffractometer operated at 50 kV and 1 mA and coupled to a Photon II detector) revealed that the best diffracting crystals were formed in a solution of 0.1 M Na HEPES pH 7.5, 10% v/v 2-Propanol, and 20% w/v Polyethylene glycol 4000.

### UDG_7 three-dimensional structure determination and refinement

The crystal structure of UDG_7 was determined by molecular replacement. X-ray diffraction data were collected on beamline ID30A-3 (MASSIF 3) at the ESRF, Grenoble, France, using an Eiger X 4M (Dectris Ltd, Baden, Switzerland) detector. A systematic grid search was performed to select the most diffracting part of each crystal. iMosflm (41) was used to calculate the strategy during data collection. All data sets were processed using the Grenoble Automatic Data ProcEssing (GrenADES) pipeline, which uses XDS (42), POINTLESS, SCALA and AIMLESS software. Data-collection statistics are given in Table S8 and the corresponding equations are the following:

$$a)\;R_{merge}=\frac{\sum \text{hkl}\sum_{i=1}^{n}\left|I_{i}\left(hkl\right)-\bar{I}\left(hkl\right)\right|}{\sum \text{hkl}\sum_{i=1}^{n}I_{i}\left(hkl\right)}$$

where I is the observed intensity, and $\bar{I}$ is the statistically-weighted average intensity of multiple observations.

$$b)\;R_{p.i.m.}=\frac{\sum_{hkl}\sqrt{1/\left(n-1\right)}\sum_{i=1}^{n}\left|I_{i}\left(hkl\right)-\bar{I}\left(hkl\right)\right|}{\sum_{hkl}\sum_{i=1}^{n}I_{i}\left(hkl\right)}$$

a redundancy-independent version of *R*_*merge*_.

$$c)\;R_{work}=\frac{\sum \text{hkl}\left|\left|F_{obs}\left(hkl\right)\right|-\left|F_{calc}\left(hkl\right)\right|\right|}{\sum \text{hkl}\left|F_{obs}\left(hkl\right)\right|}$$

where *F*_*calc*_ and |*F*_*obs*_| are the calculated and observed structure factor amplitudes, respectively. *R*_*free*_ is calculated for a randomly chosen 5% of the reflections.

The best-diffracting crystal diffracted to a resolution of 1.7 Å and belonged to the monoclinic spacegroup C2. Phaser MR was used to perform molecular replacement using the crystal structure of UDG from *M. tuberculosis* in complex with 2-thiouracil (4WS8) (43). One copy of UDG_7 was present in the asymmetric unit. The partially obtained model was completed with Buccaneer (44) and with manual modeling in COOT (45). It was then refined using REFMAC5 (46) and PDB REDO (47), interspersed with model adjustment in COOT. The final round of refinement was performed using the TLS/restrained refinement procedure, yielding a final model at 1.71 Å resolution (Protein Data Bank code 9TU4). The RMSD of bond lengths, bond angles, torsion angles, and other indicators were continuously monitored using validation tools in COOT and MOLPROBITY (48). A summary of the refinement statistics is provided in Table S8. The wwPDB Validation Service was used to validate the structures before deposition in the PDB. 3D structure figures were generated using UCSF ChimeraX (49).

### Single-site variant library (SSVL)

A single-site variant library comprising 48 amino acid positions of interest was generated for UDG_7 and ordered from Twist Bioscience. The library was cloned into the pHTP1 expression vector and transformed into *E. coli* BL21(DE3) cells as detailed above. For the first screening of the SSVL, a pooled set of 19 single-amino-acid variants for each position was expressed and purified together with wild-type UDG_7, and thermal unfolding was monitored using the thermal shift assay described above. The 16 non-catalytic thermolability hotspots identified through pooled thermal profiling were then transformed into *E. coli* BL21(DE3) cells, and for each one of 30 independent colonies, were expressed in auto-induction medium, cell lysates were prepared by chemical lysis without DNase I to preserve plasmid DNA, and each extract was subjected to an endpoint assay with uracil-containing primers designed to amplify the UDG_7 sequence before and after incubation at 35 or 45 °C. The endpoint master mix was prepared as follows: 1x NZYTaq II 2x Green Master Mix (NZYtech), 0.25 μM Primer Forward (5′-UUCCCCUCUAGAAAUAAUUUUGUUU-3′), 0.25 μM Primer Reverse (5′-CCGGAUAUAGUUCCUCCUUUC-3′), 10 μl lysate, 1 μl template DNA and water up to 50 μl. Plasmid DNA was recovered from the extracts *via* transformation into *E. coli* DH5α and substitutions were analyzed by DNA sequencing through Macrogen Europe CES service (Amsterdam, the Netherlands). The final screening of the 54 variants recovered followed the thermal shift and endpoint assays described previously.

### Statistical analysis

The experimental results were expressed as the mean of a series of replicates (n ≥ 3) and standard deviation (SD). All statistical analyses were performed using Microsoft Excel 2021 and GraphPad Prism (Version 9.0.0, Boston, MA).

## Data availability

All data presented and generated in this article is available upon a reasonable request being made to the corresponding author.

## Supporting information

This article contains supporting information.

## Conflicts of interest

The authors declare the following financial interests/personal relationships which may be considered as potential competing interests: R. S., J. T. and C. F. are employed by NZYtech, Lda., a biotechnology company dedicated to the production of enzymes.
