# Supplementary material for: Noncatalytic surface electrostatic networks tune thermolability in uracil-DNA glycosylase
Source: J Biol Chem. 2026 May 28;302(7):113212. doi: 10.1016/j.jbc.2026.113212 (PMC13316540; doi:10.1016/j.jbc.2026.113212)
Supplement: Supporting Figures [file mmc1.pdf]

Noncatalytic surface electrostatic networks tune thermolability in uracil-DNA glycosylase

Rita S. M. Simões [1, 2, 3], João S. Teodoro [1], Victor D. Alves[2, 3], Carlos M. G. A. Fontes [1, 2, 3] and **Pedro Bule [2, 3*]**

[1] NZYtech – Genes & Enzymes, Campus do Lumiar, Building J, 1649-038, Lisbon, Portugal
[2] CIISA – Centre for Interdisciplinary Research in Animal Health, Faculty of Veterinary Medicine, University of Lisbon, 1300-477, Lisbon, Portugal
[3] Associate Laboratory for Animal and Veterinary Sciences (AL4AnimalS), 1300-477, Lisbon, Portugal
* Correspondence: pedrobule@fmv.ulisboa.pt.

Supporting Information:

- **Figures S1 – S11**
- **Tables S1 – S8**

Figure S1. Maximum-likelihood phylogeny of the 24 UDGs analyzed in this study. The phylogenetic tree was generated with the NGPhylogeny.fr “one-click” workflow from a multiple sequence alignment of the 24 uracil-DNA glycosylase sequences (see Figure S2 for details). Branch lengths reflect the number of substitutions per site (scale bar). Four well-supported clades (Clusters A–D) are indicated.

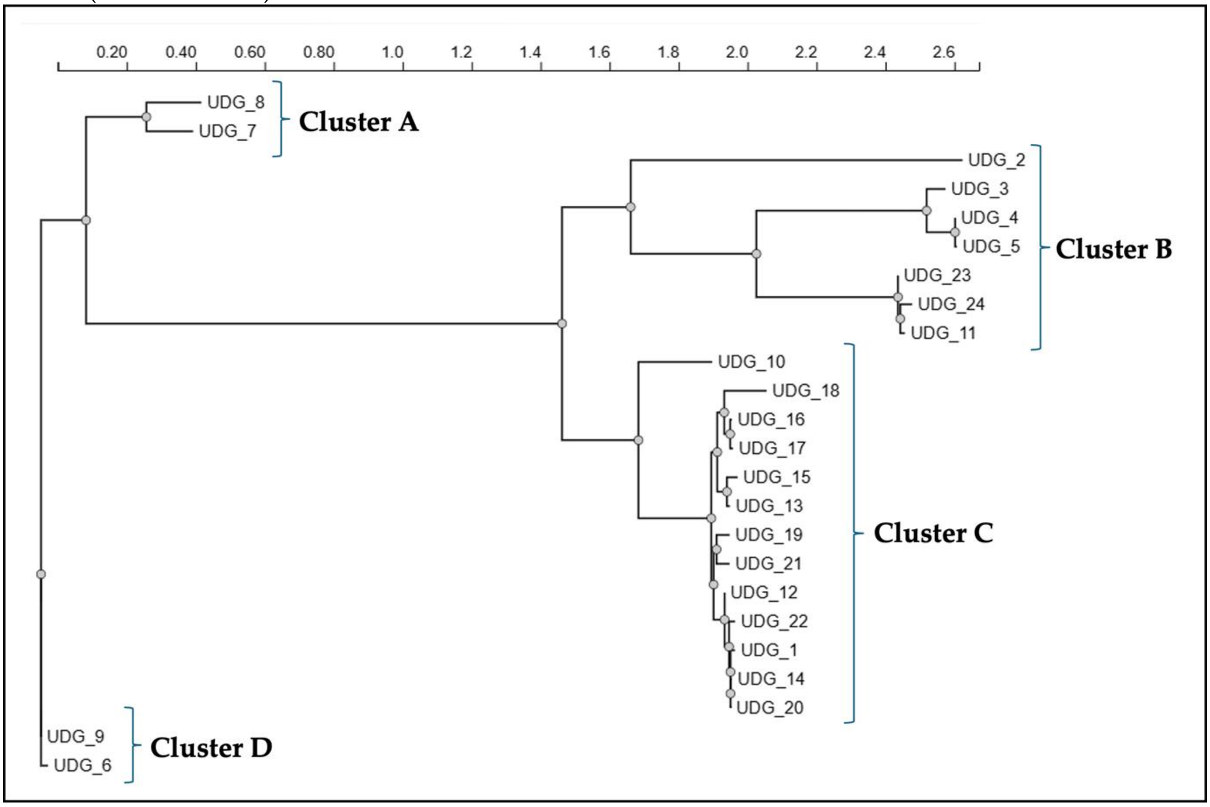

Figure S2. Multiple sequence alignment of UDG panel and conservation of canonical UDG motifs. Multiple sequence alignment of the 24 environmental UDGs (UDG_1-UDG_24), together with *E. coli* UDG (UDG_*E.coli*) and Atlantic cod UDG (UDG_Cod), is shown in three segments corresponding to the N-terminal, central and C-terminal regions of UDG_7. UDG_7 serves as the reference sequence for residue numbering. Canonical UDG motifs 1-5 are highlighted with a colored box and labeled above the alignment, and the secondary-structure elements of UDG_7 (α-helices and β-strands) are depicted as cartoons above each segment. Sequence conservation highlighting is matched with Clustal-based symbology (. : *) and sequence logos below each block report residue conservation at every position, with letter height proportional to the observed frequency. Conserved catalytic and motif residues cluster within motifs 1-5, whereas the sequence space surrounding them is more variable, providing a structural framework for engineering UDG_7 stability and thermolability.

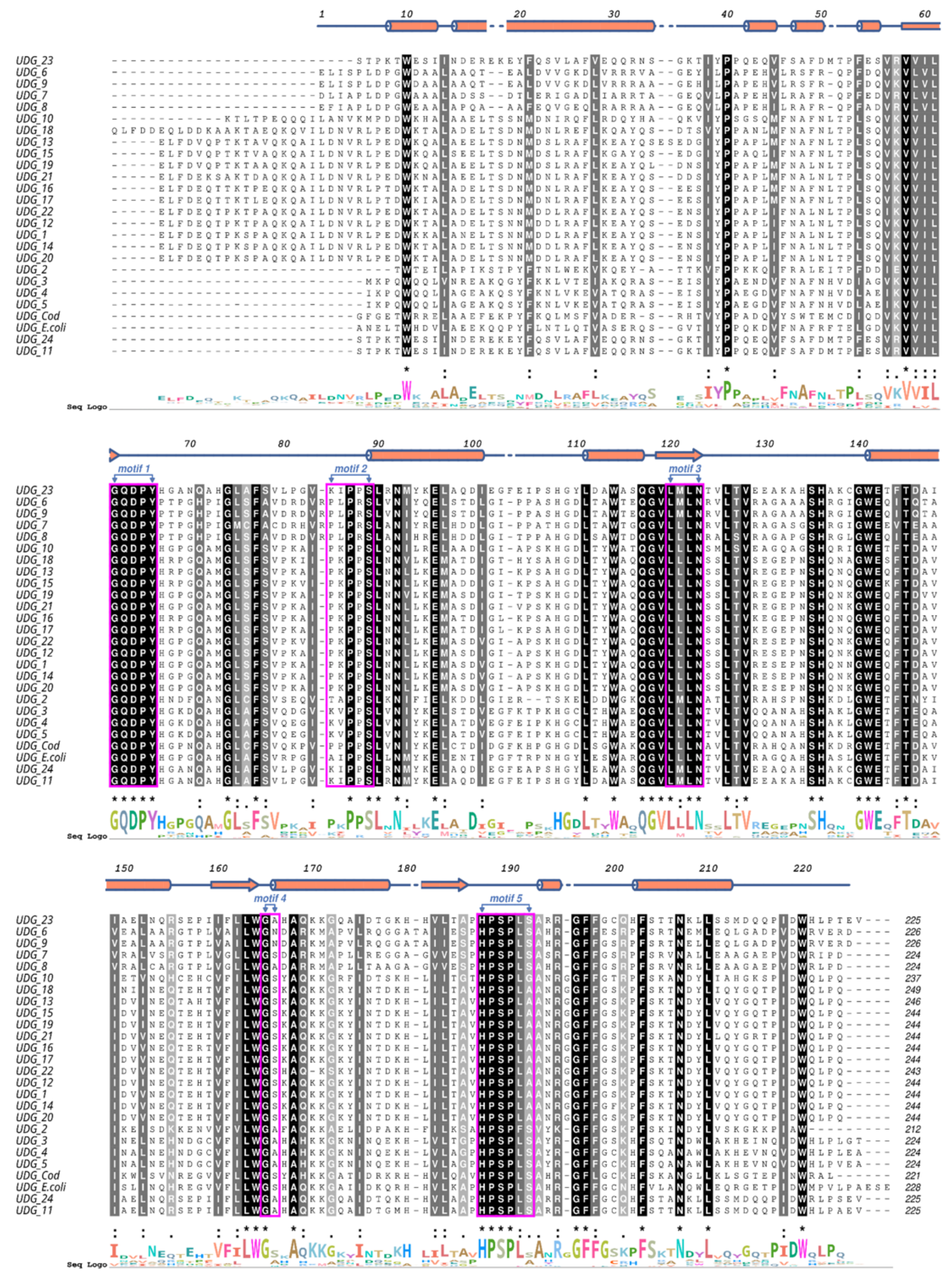

Figure S3. Representative expression and purification of recombinant uracil–DNA glycosylases in *Escherichia coli* as assessed by SDS–PAGE. Recombinant UDG homologues were expressed in *E. coli* and purified by Ni^{2+}-affinity chromatography. The gel shows 6 enzymes that accumulated in soluble form (apparent molecular mass ≈ 27 kDa) and two examples with reduced solubility (lanes 4 and 5). Lanes identified with "M" correspond to the molecular mass marker.

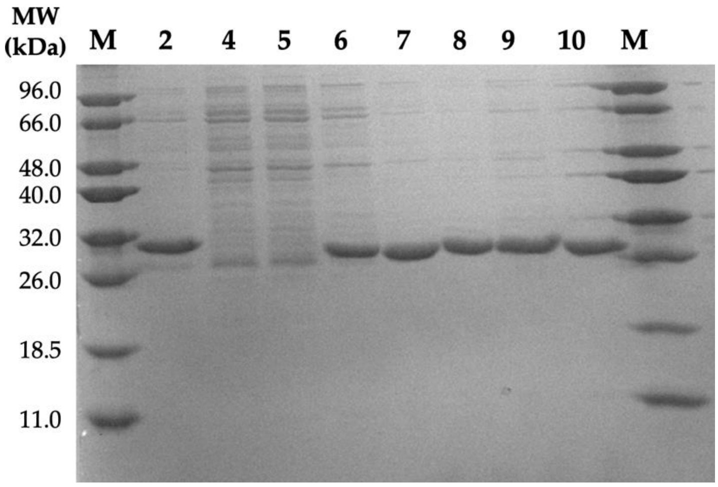

Figure S4. Activity assays at 25 ^{0}C of the 8 UDGs with best expression results. Real-time PCR assays to detect a human *PPIA* (*H. sapiens*) template after a 10-minute incubation at 25 ^{0}C with each UDG in test. Each reaction contained 10^5 copies of *hPPIA* template in which ~60% of thymine residues were replaced with uracil, together with 10^2 copies of a matched thymine-only template. For each chart the green line corresponds to Cod UDG, the orange line corresponds to the tested UDG, the black line corresponds to the no UDG control and the grey line to the no template control.

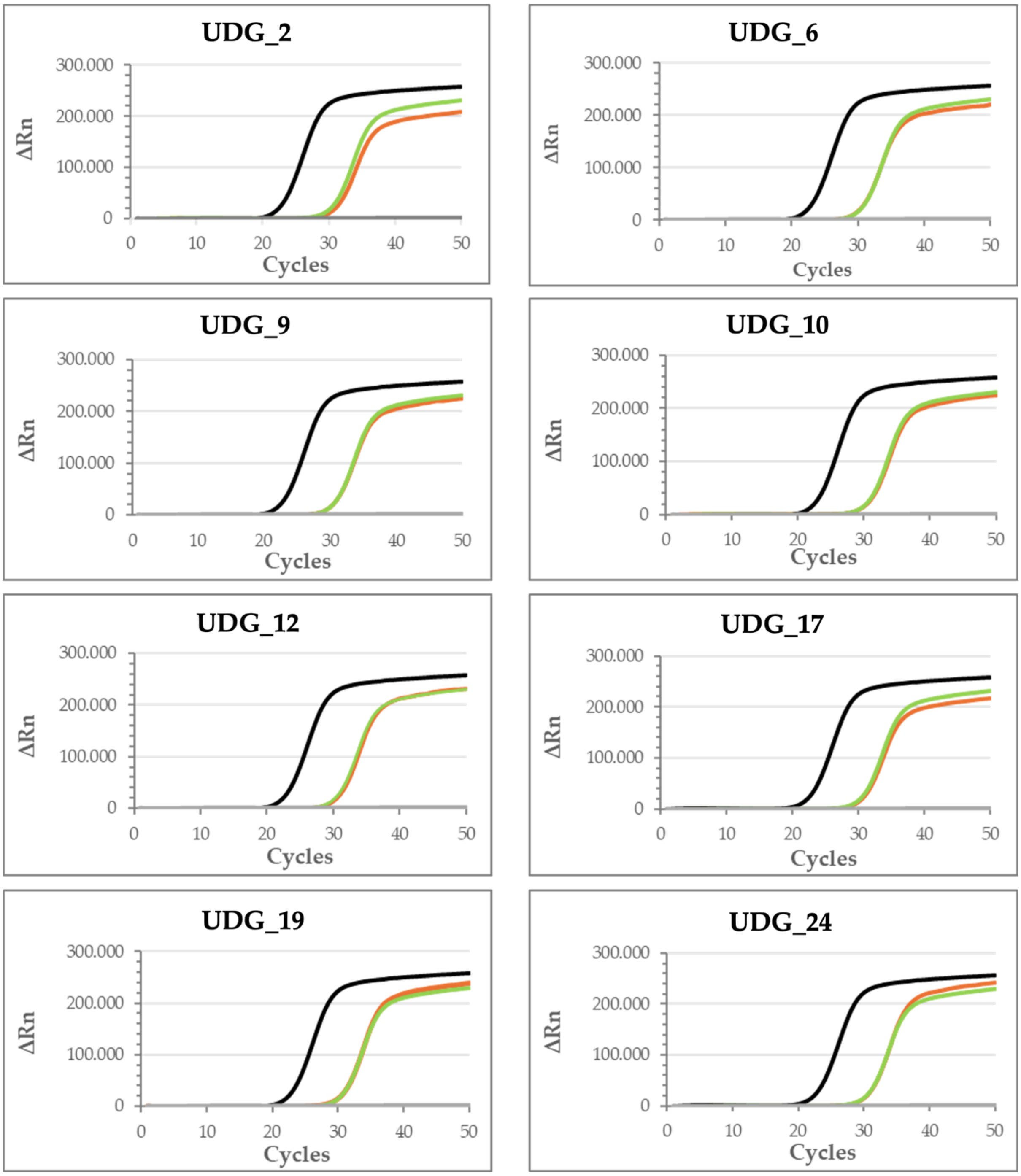

Figure S5. Inactivation assays at 50 ^{0}C of the 8 UDGs with best expression results. Real-time PCR assays to detect a human *PPIA* (*H. sapiens*) template after a 10-minute incubation at 25 ^{0}C with each UDG following a previous step in which the enzyme was inactivated for 10 minutes at 50 ^{0}C. Each reaction contained 10^5 copies of *hPPIA* template in which ~60% of thymine residues were replaced with uracil, together with 10^2 copies of a matched thymine-only template. For each chart the green line corresponds to Cod UDG, the orange line corresponds to the tested UDG, the black line corresponds to the no UDG control and the grey line to the no template control.

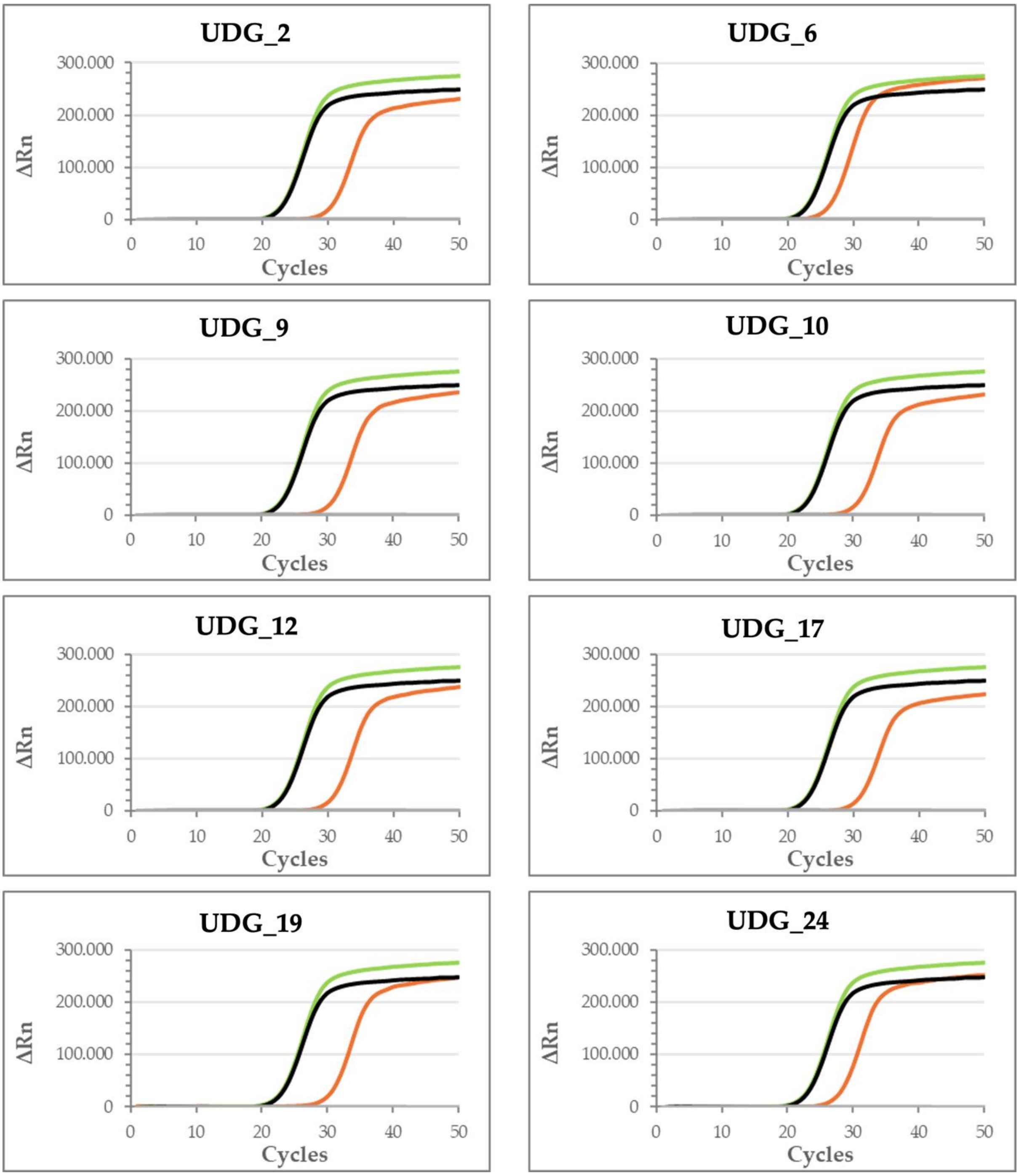

Figure S6. Benchmarking UDG_7 against Atlantic cod UDG and a commercial thermolabile UDG. (Right-side pannels) UDG_7, Atlantic cod UDG and a commercial thermolabile UDG (NEB M0372) were incubated for 2.5, 5 or 10 min at 25 ºC with 10^5 copies of *C. albicans RPR1* (top) or *H. sapiens PPIA* (bottom) templates containing 60% dUTP, supplemented with low-copy thymine-only templates. Bars show mean Ct values after qPCR amplification and error bars represent the standard deviation from replicates (n=3). The "No UDG" control corresponds to reactions in which the template mixture was not exposed to UDG. **(Left-side pannels)** The same three enzymes were pre-incubated for 10 min at the indicated temperatures (45 - 50 ºC) and then incubated at 25 ºC with 10^5 copies of *C. albicans RPR1* (top) or *H. sapiens PPIA* (bottom) templates containing 60% dUTP plus 50 copies of a thymine-only template. Bars show mean Ct values after qPCR amplification and error bars represent the standard deviation from replicates (n=3). "No UDG" denotes reactions lacking enzyme.

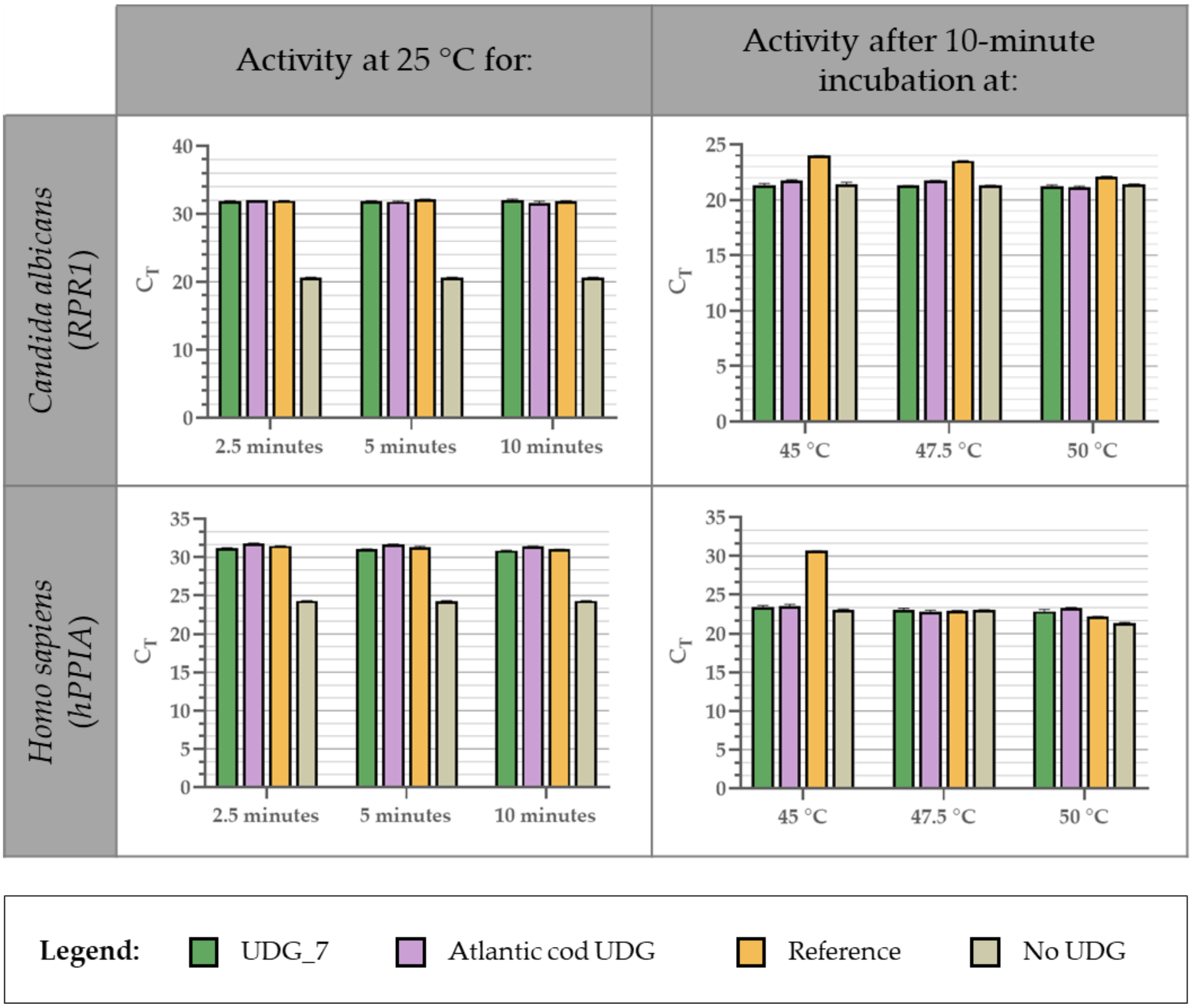

Figure S7. Active-site environment, catalytic motifs and flexibility of UDG_7. (A) Surface representation of UDG_7 (beige) with the DNA strand from the human UNG–DNA complex docked to illustrate the substrate path across the positively charged groove. The bound glycerol molecule occupying the uracil-binding pocket is shown as sticks. (B) Close-up view of the catalytic region of UDG_7 with the five conserved family-I motifs coloured as in Figure 4C (motif 1 yellow, motif 2 blue, motif 3 green, motif 4 violet, motif 5 red), emphasising the intact architecture of the active site. (C) and (D) B-factors mapped onto the molecular surface of UDG_7 in two orientations. Low-mobility regions are coloured blue and high-mobility regions red, as indicated by the scale bar. The catalytic motifs are among the coolest (most rigid) regions, whereas increased flexibility is concentrated in peripheral loops and surface patches.

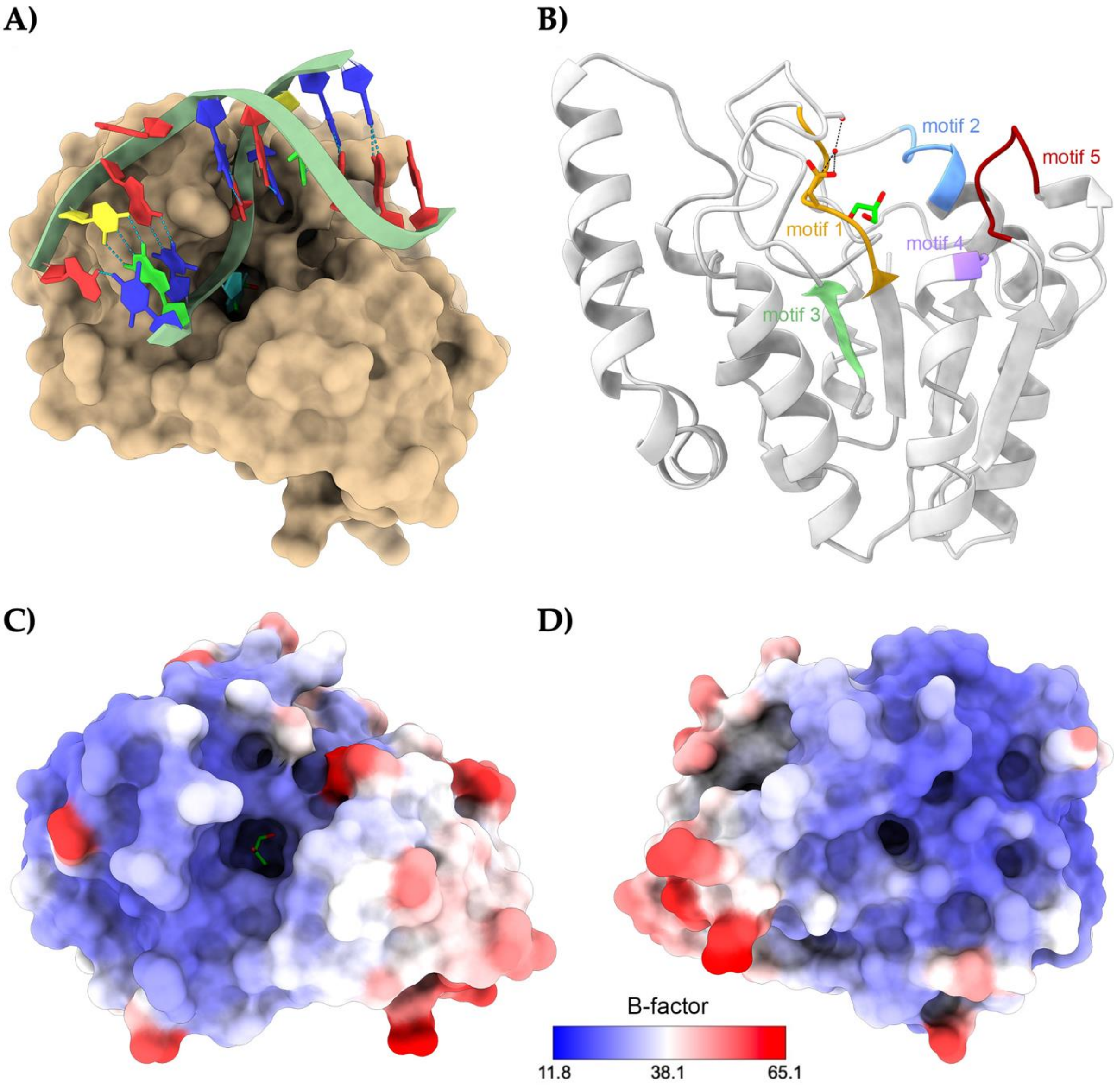

Figure S8. Global thermal profiling of pooled single-site variant libraries in UDG_7. Derivative melting curves (−dRFU/dT versus temperature) for wild-type UDG_7 (green) and pooled variant libraries (orange) at all positions that yielded soluble protein. For each targeted site, the corresponding panel shows the melting behavior of the pooled variants and the wild-type reference, together with SDS-PAGE analysis of the purified pools. Positions in which the pooled variants showed a lower Tm than UDG_7 were classified as thermolability hotspots and are summarized in Table S3.

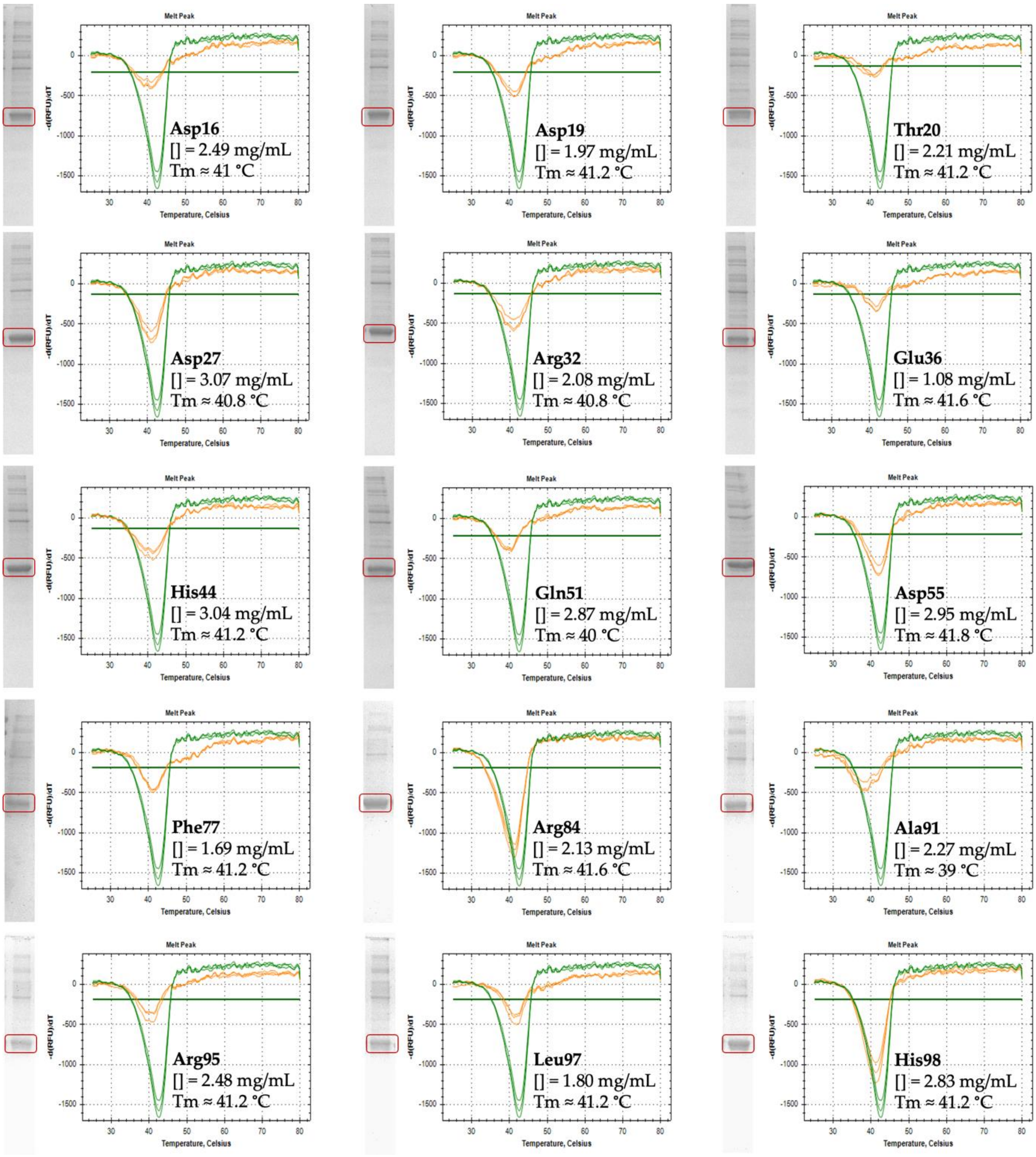

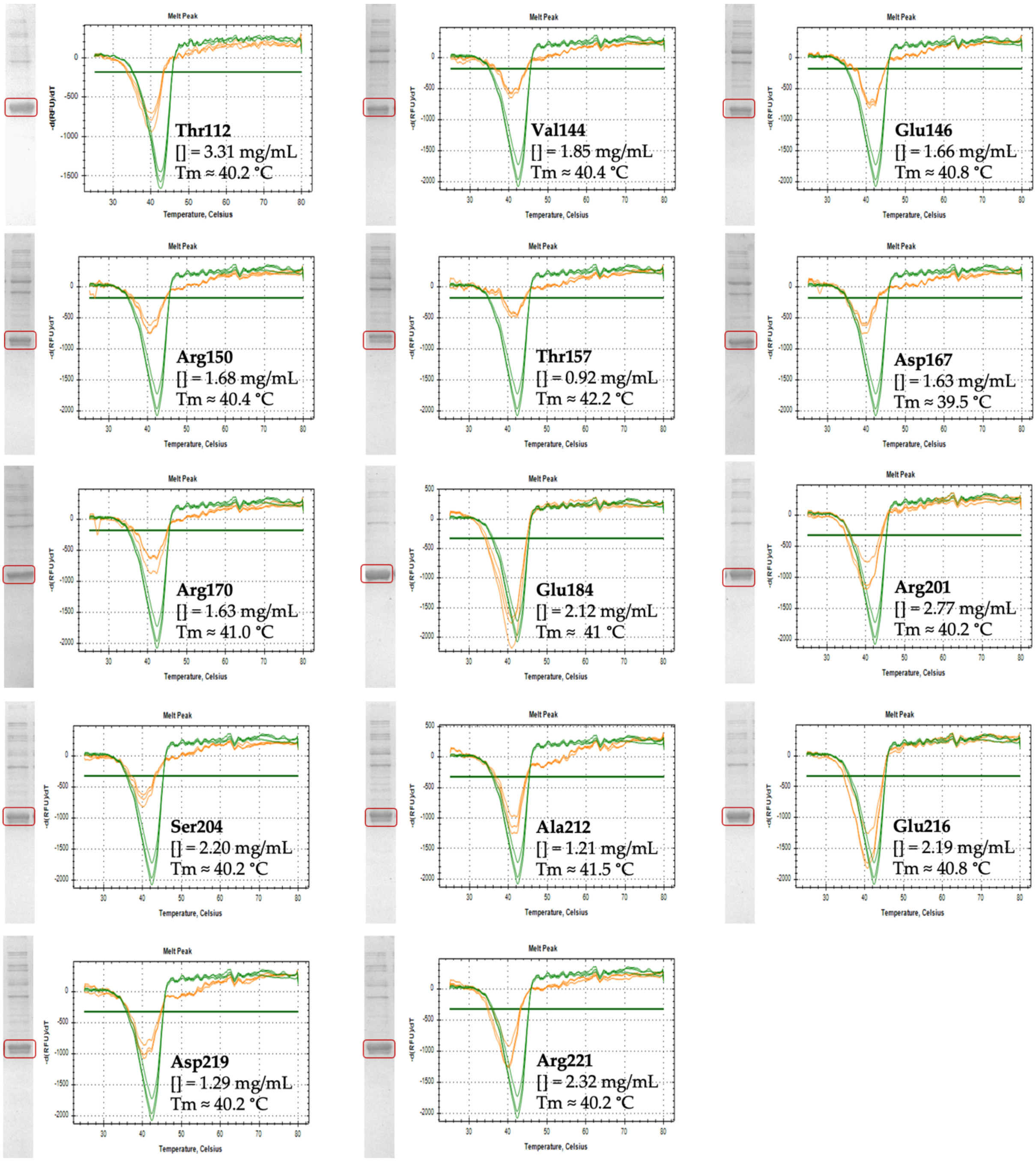
Melt Peak
-d(RFU)/dT
Temperature, Celsius
Thr112
[] = 3.31 mg/mL
Tm ≈ 40.2 °C
Val144
[] = 1.85 mg/mL
Tm ≈ 40.4 °C
Glu146
[] = 1.66 mg/mL
Tm ≈ 40.8 °C
Arg150
[] = 1.68 mg/mL
Tm ≈ 40.4 °C
Thr157
[] = 0.92 mg/mL
Tm ≈ 42.2 °C
Asp167
[] = 1.63 mg/mL
Tm ≈ 39.5 °C
Arg170
[] = 1.63 mg/mL
Tm ≈ 41.0 °C
Glu184
[] = 2.12 mg/mL
Tm ≈ 41 °C
Arg201
[] = 2.77 mg/mL
Tm ≈ 40.2 °C
Ser204
[] = 2.20 mg/mL
Tm ≈ 40.2 °C
Ala212
[] = 1.21 mg/mL
Tm ≈ 41.5 °C
Glu216
[] = 2.19 mg/mL
Tm ≈ 40.8 °C
Asp219
[] = 1.29 mg/mL
Tm ≈ 40.2 °C
Arg221
[] = 2.32 mg/mL
Tm ≈ 40.2 °C

Figure S9. Representative plate layout of the three-temperature functional screen of UDG_7 single-site variants. Representative agarose gel analysis of the three-step screen used to identify thermolabile, catalytically competent UDG_7 variants at selected SSVL positions. For each hotspot, 30 individual clones were expressed in *E. coli* BL21 and tested in parallel. "Activity screening at 25 ⁰C" shows UDG activity at the operating temperature of the assay, whereas "Inactivation at 35 ⁰C" and "Inactivation at 45 ⁰C" show residual activity after pre-incubation of the enzyme at the indicated temperature. The presence or absence of the amplicon band reports on UDG-dependent degradation of the uracil-containing primers, as detailed in Experimental Procedures. Lanes 1–30 correspond to individual clones, lane "C" is the UDG_7 wild-type control and lane "NC" is the no-enzyme control. Clones boxed in green exemplify variants that retain activity at 25 ⁰C but lose detectable activity after pre-incubation at 35 ⁰C and 45 ⁰C ("on–off–off" phenotype). Full data is compiled in Table S5.

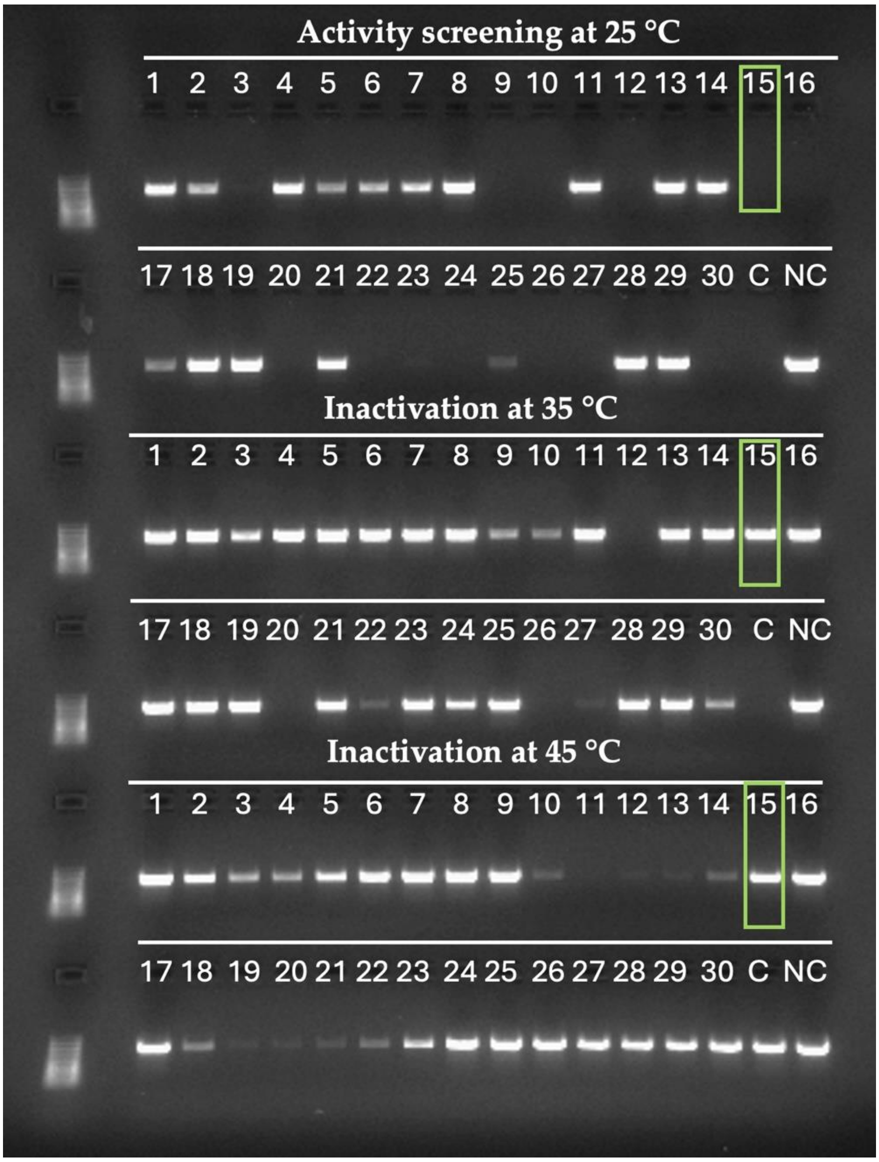

Figure S10. Purification of 54 thermolabile UDG_7 variants selected from the hotspot screen. SDS–PAGE analysis of representative UDG_7 variants recovered from the high-throughput hotspot screen. Each panel shows purified single-site mutants (lanes labelled above the gels) alongside molecular-weight markers. All variants migrate as a single major band at ~27–30 kDa, consistent with the predicted molecular mass of UDG_7, and show minimal detectable contaminants and molecular integrity, indicating preparation suitable for biophysical and kinetic characterization (see Table S6).

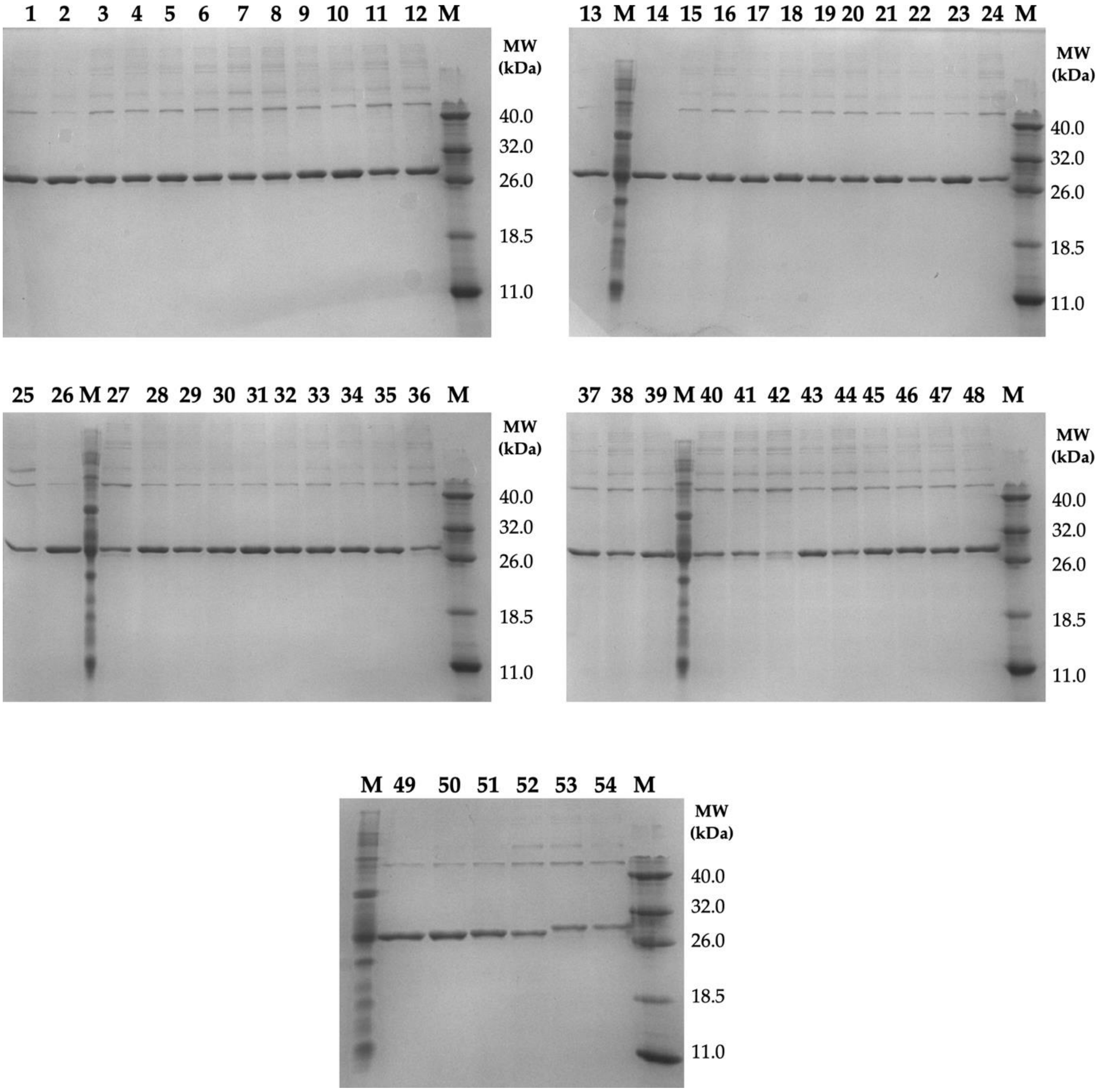

Figure S11. Thermal unfolding profiles of individual UDG_7 hotspot variants. Fluorescence-based melting curves for purified UDG_7 variants carrying single-amino-acid substitutions at SSVL-defined hotspot positions. For each variant, the first derivative of the fluorescence signal with respect to temperature (−dF/dT) is plotted as a function of temperature, and the apparent melting temperature (Tm) is indicated on the panel (see Table S6 for abbreviated information).

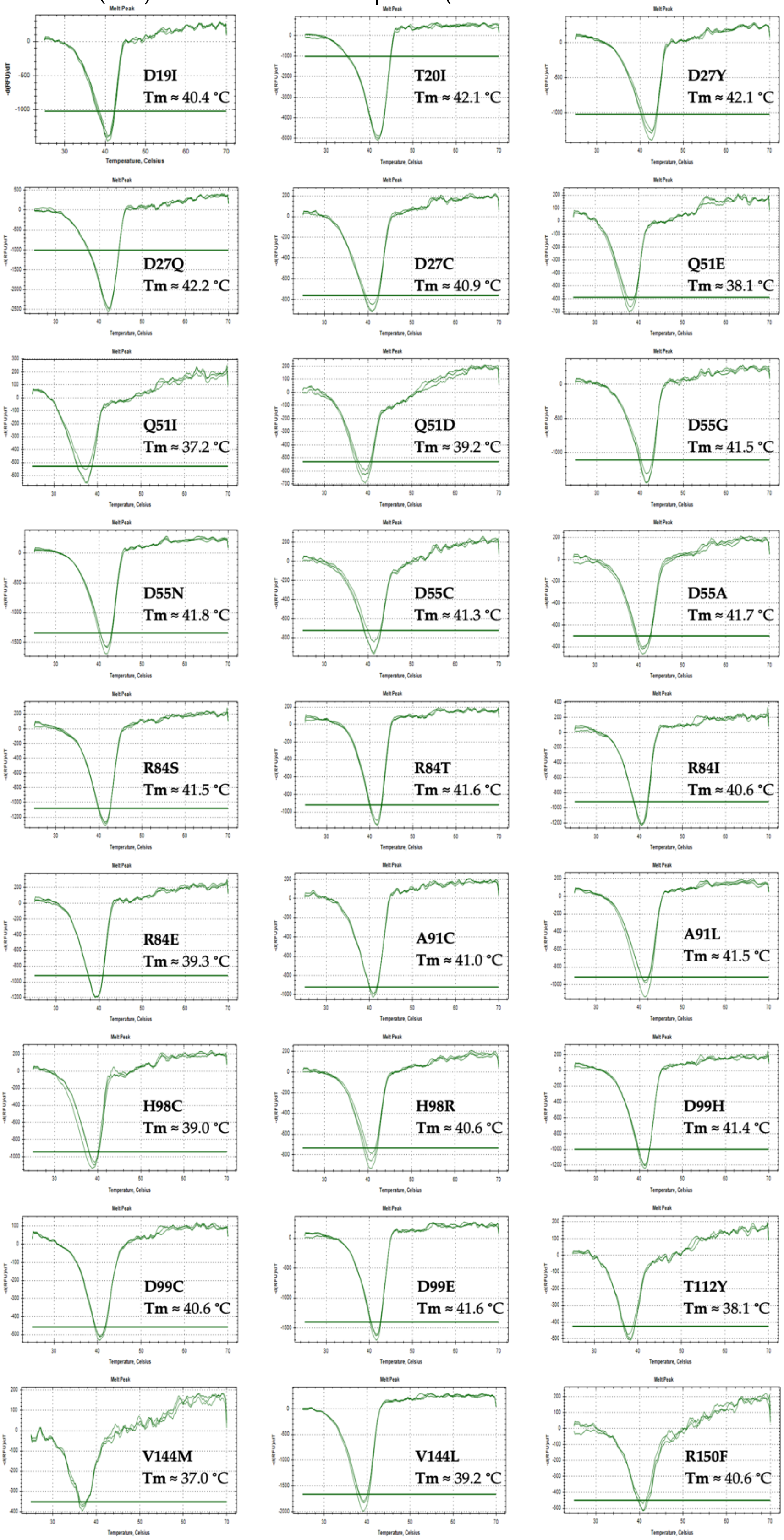

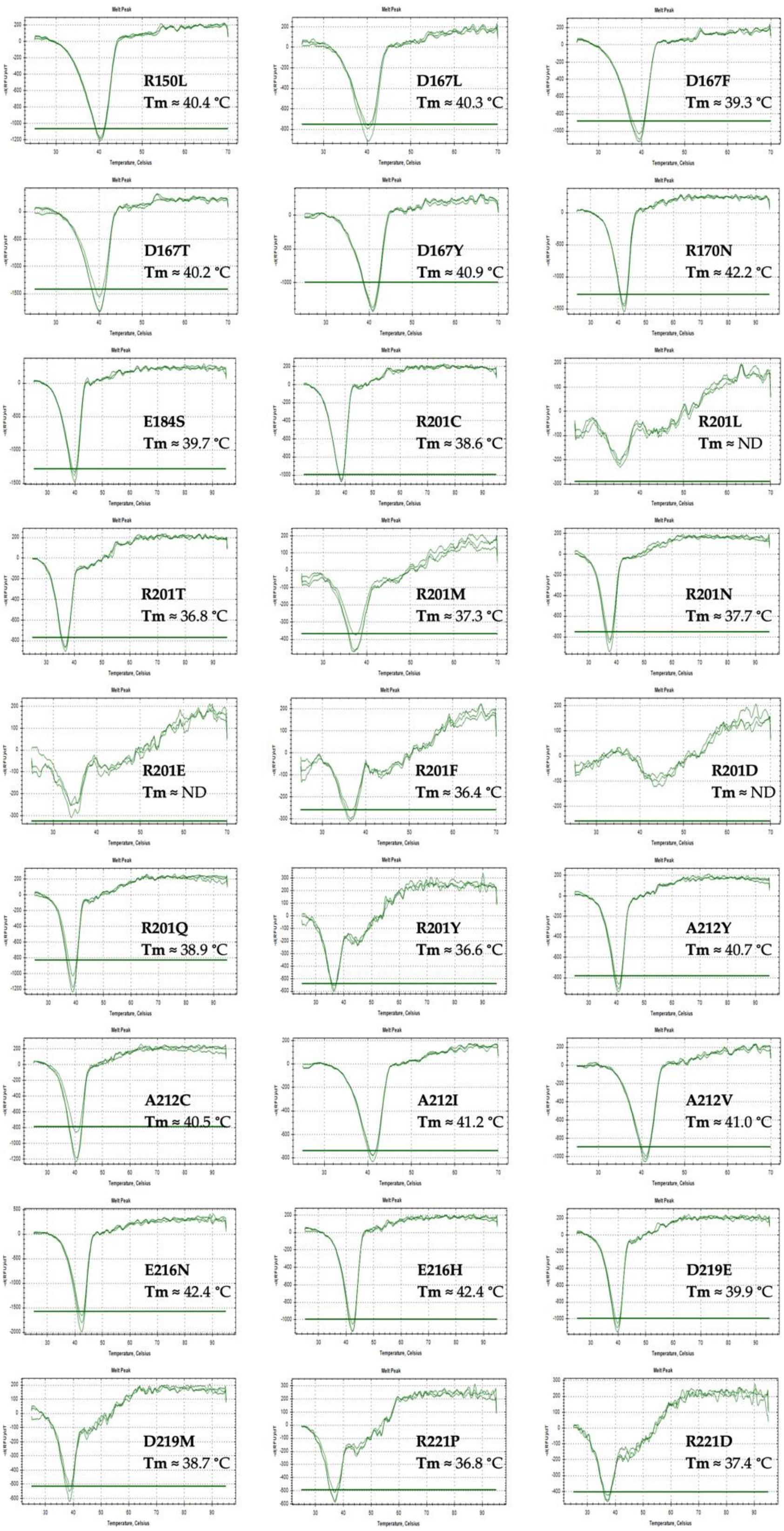
R150L
Tm ≈ 40.4 °C
D167L
Tm ≈ 40.3 °C
D167F
Tm ≈ 39.3 °C
D167T
Tm ≈ 40.2 °C
D167Y
Tm ≈ 40.9 °C
R170N
Tm ≈ 42.2 °C
E184S
Tm ≈ 39.7 °C
R201C
Tm ≈ 38.6 °C
R201L
Tm ≈ ND
R201T
Tm ≈ 36.8 °C
R201M
Tm ≈ 37.3 °C
R201N
Tm ≈ 37.7 °C
R201E
Tm ≈ ND
R201F
Tm ≈ 36.4 °C
R201D
Tm ≈ ND
R201Q
Tm ≈ 38.9 °C
R201Y
Tm ≈ 36.6 °C
A212Y
Tm ≈ 40.7 °C
A212C
Tm ≈ 40.5 °C
A212I
Tm ≈ 41.2 °C
A212V
Tm ≈ 41.0 °C
E216N
Tm ≈ 42.4 °C
E216H
Tm ≈ 42.4 °C
D219E
Tm ≈ 39.9 °C
D219M
Tm ≈ 38.7 °C
R221P
Tm ≈ 36.8 °C
R221D
Tm ≈ 37.4 °C

Supporting Tables are too large and have therefore been submitted separately in .xlsx format:

Table S1. Origin and ecological context of the UDG panel. For each uracil-DNA glycosylase (UDG_1-UDG_24), the table lists the source microorganism, GenBank accession number of the corresponding protein sequence, the typical ecosystem from which the organism is isolated, its reported thermal class (psychrophile, psychrotroph, mesophile, or eurythermal/psychrotolerant), and the characteristic growth temperature range and/or optimum. These annotations provide the environmental and physiological background used to assemble a diversity-guided panel spanning cold-adapted and mesophilic UDGs.

Table S2. Sequence-derived physicochemical properties and soluble yields of the UDG panel. For each uracil-DNA glycosylase (UDG_1-UDG_24), as well as the reference enzymes from *E. coli* (UDG_*E. coli*) and Atlantic cod (UDG_Cod), the table lists the theoretical molecular weight (MW, kDa), molar extinction coefficient (ε, $M^{-1}\cdot cm^{-1}$), and isoelectric point (pI) calculated from the amino-acid sequence, together with the experimentally determined yield of soluble protein after Ni^{2+}-affinity purification under standard expression conditions (Yield, $mg\cdot L^{-1}$ culture). "Insoluble" indicates that the corresponding construct was produced predominantly in the insoluble fraction and could not be purified in sufficient yield for further characterization.

Table S3. Structure-guided annotation of the UDG_7 single-site variant library (SSVL) and stabilizing interaction context. Annotations were derived from the UDG_7 crystal structure (PDB 9TU4, Chain A). The table reports SSVL index, native UDG numbering (UDG position = PDB residue − 19) and PDB residue numbering, the wild-type residue, and a stability-oriented structural classification. Structural environment and Structural role summarize local packing and the most likely stabilizing function at each site; secondary-structure assignments (helix/β-strand/loop) follow Figure 5. Dominant contacts list a curated subset of stabilizing interactions (salt-bridge clusters, hydrogen bonds, and backbone scaffold participation) prioritized by distance and network context; weak H-bonds denote contacts >3.5 Å and ≤4.0 Å. The complete heavy-atom contact inventory (≤4.0 Å) is provided in All contacts for traceability. Long-range tags indicate sequence-distant interactions ($|\Delta i| > 20$), highlighting potential inter-segment coupling relevant to thermolability. Protein yield (mg/L) corresponds to the concentration of pooled variants obtained after Ni^{2+}-affinity purification and is used as a qualitative proxy for soluble expression; Insoluble indicates that soluble material was not recovered under these conditions. Apparent melting temperatures (Tm) were derived from fluorescence-based thermal unfolding curves for each pooled variant set; ND indicates that no reliable transition could be assigned. Peak definition qualitatively scores the sharpness and interpretability of the main unfolding transition (Excellent, Good, Low, or ND). No gene denotes positions for which the designed variant cassette was not recovered after cloning and NGS and therefore could not be analyzed. Thermolability hotspot indicates positions classified as hotspots in this study (Yes/No).

Table S4. Deep-sequencing composition of the UDG_7 single-site variant library (SSVL). For each targeted SSVL position in UDG_7, the table lists the corresponding residue number in the UDG_7 sequence ("UDG position"), the wild-type (WT) codon and amino acid, and all observed variant codons and encoded residues obtained after cloning of the pooled library. "Variant proportion" indicates the percentage of sequencing reads for each codon at that position, expressed as a fraction of all reads mapping to that site. Numbers colored in red indicate percentages below 2%. "Variant sum/position" reports the cumulative percentage of variant reads per position and reflects overall coverage and representation of the 19 non-wild-type amino acids

at that site. Together, these data document the composition and near-saturation of the UDG_7 SSVL used for functional and thermal profiling.

Table S5. Three-temperature screening outcome for UDG_7 single-site variant libraries with activity and thermostability measured as exemplified in Figure S8. Summary of the high-throughput functional screen performed on individual clones derived from the 16 SSVL hotspot positions. For each SSVL position (rows), 30 clones (columns 1–30) were tested for activity at 25 °C and for residual activity after pre-incubation at 35 °C and 45 °C. Green check marks (✓) indicate successful activity or inactivation, whereas red crosses (✗) indicate the opposite. Thus, each clone is scored across three temperatures as active or inactive according to the gel pattern shown in Figure S8. The rightmost column reports, for each SSVL position, the number of clones that meet the selection criterion of retaining activity at 25 °C while losing detectable activity at both 35 °C and 45 °C ("on–off–off" phenotype). These clones were selected for further characterization.

Table S6. Sequence and thermal properties of thermolabile UDG_7 variants recovered from the hotspot screen. Summary of single-site UDG_7 variants isolated from the high-throughput hotspot screen. For each position in the single-site variant library (SSVL Position and corresponding UDG_7 Position), the wild-type (WT) residue, the mutant residue, and the number of times that substitution was independently recovered (# Clones) are shown. Average Tm values were determined from fluorescence-based melting assays on individually purified proteins (see Experimental Procedures) and are reported in °C (see Figure S10). "ND" indicates that no reliable cooperative melting transition could be assigned. Wild-type UDG_7 displays a Tm of ~42.5 °C under the same conditions.

Table S7. Primer and probe sequences for each target. For the two targets selected for real-time PCR assays - *PPIA* gene (*H. sapiens*) and *RPR1* gene (*C. albicans*) - a set of primers and probe was designed.

Table S8. X-ray diffraction data collection and refinement statistics. The crystal structure of UDG_7 was determined by molecular replacement. X-ray diffraction and corresponding data were collected. Values in parentheses are for the highest resolution shell.
